# Concurrent circulation of avian influenza viruses H5N1 and H9N2 enhances the genetic evolution of reassortant viruses in Egyptian poultry populations

**DOI:** 10.1371/journal.pone.0348609

**Published:** 2026-05-08

**Authors:** Nahed Yehia, Mahmoud Ibrahim, Rawan Muhammad Shady, Ahmed Abd Elhalem Mohamed, Dalia Said, Mohamed E. Taha, Abdelsatar Arafa, Samah Eid, Mohamed A. Shalaby, Uwe Truyen, Rea Maja Kobialka, Ahmed Abd El Wahed, Arianna Ceruti

**Affiliations:** 1 Reference Laboratory for Veterinary Quality Control on Poultry Production, Animal Health Research Institute, Agriculture Research Center, Dokki, Giza, Egypt; 2 Birds and Rabbit Medicine Department, Faculty of Veterinary Medicine, Sadat City University, Menoufiya, Egypt; 3 Egyptian Company for Biological & Pharmaceutical Industries (Vaccine Valley), October City, Giza, Egypt; 4 Department of Food Hygiene, Safety and Technology, Faculty of Veterinary Medicine, Badr University in Cairo (BUC), Cairo, Badr City, Egypt; 5 Department of Virology, Faculty of Veterinary Medicine, Cairo University, Cairo, Egypt; 6 Institute of Animal Hygiene and Veterinary Public Health, Leipzig University, Leipzig, Germany; University of South Dakota, UNITED STATES OF AMERICA

## Abstract

The co-circulation of the recently emerged H5N1 clade 2.3.4.4b and the endemic H9N2 avian influenza viruses (AIV) in poultry farms has led to significant economic losses and increased the likelihood of viral reassortment. Continuous and extensive surveillance with full genome sequencing is highly recommended. The objective of this study was to monitor AIV circulating in Egyptian poultry populations throughout 2024 using molecular surveillance and to detect genetic reassortment events. A total of 50 chicken flocks that exhibited respiratory symptoms from seven governorates in Egypt were tested for avian influenza H5, H9, Infectious Bronchitis virus (IBV), and Newcastle Disease Virus (NDV) using real-time RT-PCR. Four flocks that tested positive for H5 (AN1, AN6, AN7, and AN8) and three flocks that tested positive for H9N2 (AN2, AN3, and AN4) were selected for isolation and full-genome sequencing. They were subjected to virus isolation in specific-pathogen-free (SPF) embryonated chicken eggs, and identification was done using real-time RT-PCR assay. The full-genome sequencing was performed using rapid barcoding from Oxford Nanopore Technologies. The genome analysis revealed a H5N2 reassortant virus, comprising the HA, PB2, PB1, and PA gene segments from H5N1 clade 2.3.4.4b (EA-2021-AB), while the NA, NP, NS, and M genes were from H9N2 (G5.6). Additionally, two reassorted H9N2 viruses were identified, containing HA, NA, NP, M, and NS genes from H9N2 (G5.6), and PB1, PB2, and PA genes from Highly Pathogenic Avian Influenza H5N1 virus Clade 2.3.4.4b (EA-2021-AB). Interestingly, both reassortant H9N2 viruses have specific adaptive mutations in some of their internal genes that were not present in any other Egyptian H9N2 viruses. Several mutations, potentially associated with increased virulence and mammalian adaptation, were also detected in the internal genes. This study highlights the emergence of novel reassortant AIV viruses and underscores the need for continuous molecular surveillance, as well as further studies on the pathogenicity and vaccine efficacy against these newly emerged viruses.

## Introduction

Influenza viruses are contagious respiratory pathogens that affect birds, humans, and other animals. Belonging to the *Orthomyxoviridae* family, these viruses, especially Influenza A, can evolve rapidly through mutations and reassortment, leading to new viruses with pandemic potential. Avian influenza viruses (AIV), such as H5 and H7 subtypes, pose significant risks to poultry and public health, making continuous monitoring and control efforts essential [1]. Major economic losses due to AIV outbreaks have been described across many countries, substantially impacting regional economies and livelihoods [2–5]. The AIV has eight gene segments encoding several structural and non-structural proteins, each playing a distinct role in the viral lifecycle, host interaction, and pathogenicity. The surface glycoproteins hemagglutinin (HA) and neuraminidase (NA) are responsible for viral attachment to host cells and release of progeny virions, respectively. HA mediates binding to sialic acid receptors and membrane fusion, while NA cleaves sialic acid residues to facilitate viral spread [6]. The PB2, PB1, and PA genes encode the RNA-dependent RNA polymerase complex, essential for transcription and replication of the viral genome. PB2 contributes to host adaptation, PB1 catalyzes RNA synthesis, and PA possesses endonuclease activity required for cap-snatching [7,8]. NP (nucleoprotein) binds viral RNA, forming ribonucleoprotein complexes critical for genome replication and nuclear trafficking. The M segment encodes M1, which supports virion structure and assembly, and M2, an ion channel protein involved in viral uncoating [9,10]. The NS segment encodes NS1, a key interferon antagonist that suppresses host antiviral responses, and NS2/NEP, which mediates nuclear export of viral RNPs [11]. Together, these eight genes orchestrate efficient viral replication, immune evasion, host specificity, and evolution through reassortment, thereby contributing to the emergence of novel viruses with pandemic potential [12,13].

Reassortant avian influenza viruses arise when two or more influenza viruses co-infect a single host and exchange gene segments, potentially leading to novel viruses with altered pathogenicity, host range, and transmissibility. This genetic shuffling is particularly concerning when low pathogenic avian influenza (LPAI) viruses acquire internal genes from highly pathogenic viruses or from viruses adapted to mammals, which can enhance their zoonotic and pandemic potential [14]. To detect reassortant viruses accurately and rapidly, whole-genome sequencing techniques such as next-generation sequencing (NGS) are used [15]. Integrating single-reaction RT-PCR amplification of all influenza genome segments with the strengths of Oxford Nanopore Technologies sequencing (ONT) enables a rapid and reliable sequencing strategy that has been successfully applied to influenza A virus surveillance [16]. This is usually followed by phylogenetic analysis to trace the origin of each gene segment. Continuous surveillance and molecular characterization of circulating viruses are therefore essential for early identification of potentially dangerous reassortants and for guiding appropriate control strategies [17].

The H9N2 virus was first detected in Egypt in 2011, which phylogenetically clustered within the G1 lineage (group B) based on the HA gene sequence [18]. The virus became endemic within a short period of time across the country, infecting different poultry species reared in Egypt [19]. Despite the co-circulation with the H5N1 clade 2.2.1 at the same time [20], no report for reassortment between both AIV subtypes in Egypt was described. However, another study reported two H5N1 clade 2.3.2.1 reassortants with H9N2 in Bangladesh [21]. Reports of reassortment events for the LPAI H9N2 virus with other AIV subtypes were previously recorded. The originally introduced H9N2 virus was known as genotype I and circulated from 2010 to 2013. In 2014, novel H9N2 reassortant viruses were detected in pigeons that acquired five gene segments from LPAIV different subtypes (PB2, PB1, PA, NP and NS) and designated as genotype II [22]. Further reassortment occurred with new H9N2 reassortants (genotype III) reported from chickens and quails, which acquired the PB2, PB1, PA, and NS gene segments from Eurasian low pathogenic AIVs [23].

The Highly Pathogenic Avian Influenza (HPAI) H5N8 virus clade 2.3.4.4B was introduced into Egypt through migratory wild birds in late 2016 [24]. Since then, repeated detections of H5N8 AIV in domestic poultry flocks across the country have been reported, and the virus became endemic within a short period [25]. The H5N8 AIV has replaced the previously circulating H5N1 clade 2.2.1, and since 2017, H5N8 and H9N2 AIVs have co-circulated in Egyptian domestic poultry. In December 2018, a novel reassortant HPAI H5N2 virus was detected in a commercial Muscovy duck farm due to reassortment between H5N8 and H9N2, with only one gene segment exchange (NA from H9N2 introduced into H5N8) [26]. Another novel H5N2 reassortant was detected in chicken during 2019, where the H9N2 acquired the HA gene segment from the HPAI H5N8 virus [27]. Previously, reports for the repeated emergence of natural reassortants between H9N2 viruses and H5 HPAI viruses, with H5N2 also emerging, have been observed in Southeast Asia [28]. This indicates the high tendency of these HPAI H5 viruses of clade 2.3.4.4 to reassort with not only the co-circulating H9N2 as reported in Egypt but also with various influenza A viruses of wild birds or poultry [29,30]. Additional reassortants between H5N8 and H9N2 AIVs in Egyptian poultry are considered as likely.

Recently, the HPAIV H5N1 clade 2.3.4.4b was introduced into Egypt in 2021, which takes place of the previous HPAI H5N8 clade 2.3.4.4b viruses [31]. The circulation of this H5N1 AIV in endemically infected poultry populations with the LPAIV H9N2 and the high ability of both subtypes for reassortment pose a high risk for poultry and humans. Indeed, a novel reassortant HPAI H5N2 virus was detected in ducks in June 2024 due to reassortment between the A (H5N1) clade 2.3.3.4b virus and H9N2 G1-like virus [31]. Previous studies reported the high genetic diversity of the circulating A (H5) viruses of clade 2.3.4.4b in Europe during 2020–2022, with frequent reassortment events [32], leading to the emergence of a new genotype with gull-adapted genes that became endemic in the wild bird population of Europe. Moreover, extensive reassortment events between different genotypes of H5N8 and H5N1 clade 2.3.4.4b or with different LPAIV subtypes have been previously reported in USA [7], China [33], South Korea [34,35], and Bangladesh [21].

The high tendency of the HPAI H5N1virus of clade 2.3.4.4b for reassortment, a wide range of host species, including mammals, and the ability to acquire adaptive mutations make the prediction of its evolution and spread challenging. Therefore, continuous and extensive surveillance with full genome sequencing is highly recommended. To address this issue, this study aimed to monitor avian influenza viruses in Egyptian poultry during 2024, using ONT to rapidly identify reassortment events and virulence mutations through full genome analysis.

## Materials and methods

### Clinical samples

Tracheal swabs or tracheal tissue were collected from 50 chicken flocks (30 backyard and 20 broiler farms) during routine surveillance efforts. Clinical symptoms were associated with severe respiratory distress, swollen heads, and decreased egg production and mortality ranged from 7–80%. The samples were received from seven governorates during 2024 (S1 Table).

### RNA extraction and real-time RT-PCR for initial screening

RNA extraction was done using the QIAamp Viral RNA Mini Kit (Qiagen, Hilden, Germany), viral RNA was isolated from clinical samples in compliance with the manufacturer’s instructions. For the extraction process, 200 microliters of tracheal swab suspensions, allantoic fluid and phosphate-buffered saline (PBS) were utilized. The RNA was eluted in a final volume of 50 µL and then stored at −80 °C.

Five swabs per flock and/or five tracheal tissue homogenates from dead chickens from affected flocks were collected and screened by real-time RT-PCR for H5, H9, NDV, and IBV using primers and probes specific to H5 HA gene, H9 HA gene, NDV F gene, and IBV N gene, respectively (S2 Table). Reference strains were used as positive controls. One-step RT-PCR was carried out using AgPath-ID™ One-Step RT-PCR (Thermo Scientific, USA) and performed by using StepOnePlus Real-Time PCR machine (Applied Biosystem Thermo scientific, USA).

### Virus isolation

The flocks that tested positive for one AIV subtype, either H5 or H9, were isolated in Specific Pathogen-Free Embryonated Chicken Eggs (SPF-ECE). The eggs were then candled daily for up to three days to check embryo viability. The allantoic fluids were collected and tested for HA activity following World Animal Health Organization protocol [36]. Subsequently, the allantoic fluids were tested by real-time RT-PCR for H5, H9, NDV, and IBV for purity confirmation.

### Whole genome sequencing

Seven isolates with single infection and H5 or H9 detection from different governorates were used for whole genome sequencing (Table 1).

**Table 1:** The origin of the seven AIV chicken isolates sequenced in this study.

Isolate* Code	Subtype	Governorate
AN1	H5N1	Assiut
AN2	H9N2	Dakahlia
AN3	Re H9N2	Kafr El Sheikh
AN4	Re H9N2	Gharbiya
AN6	Re H5N2	Aswan
AN7	H5N1	Qena
AN8	H5N1	El-Wadi El-gadid

*All viruses are isolated from chickens. Re= Reassortant.

### Nucleic acid extraction and RT-PCR of isolates

The nucleic acids of the seven isolate were extracted using Dynabeads SILANE Viral NA Kit (Thermo Fisher Scientific Inc, Waltham, Massachusetts, United States). A RT-PCR was performed using two universal primers for influenza A virus

(MBTuni-12:[5′-ACGCGTGATCAGCAAAAGCAGG-3’] and

MBTuni-13: [5′-ACGCGTGATCAGTAGAAACAAGG-3’]) [37].

The SuperScript III One-Step RT-PCR System with Platinum™ Taq High Fidelity DNA-Polymerase kit (Thermo Fisher Scientific Inc, Waltham, Massachusetts, United States) was used with the following conditions: reverse transcription step at 42 °C for 60 minutes, denaturation step at 94 °C for 2 minutes. The amplification occurs in two phases: first, 5 cycles with 94 °C for 30 seconds, 45 °C for 30 seconds, and 68 °C for 3 minutes, followed by 31 cycles with 94 °C for 30 seconds, 57 °C for 30 seconds, and 68 °C for 3 minutes.

Subsequently, a purification protocol based on AMPure Agent Court beads (Beckman Coulter, Brea, California, United States) was performed according to the manufacturer’s instructions. The purified DNA was used for downstream analysis.

### Oxford nanopore sequencing

The metagenomic analysis of the samples was performed using Oxford Nanopore Sequencing (ONT). The purified nucleic acids were prepared using the Rapid sequencing DNA V14 - barcoding kit (SQK-RBK 114.24, Oxford Nanopore Technologies, Oxford, United Kingdom). The library preparation was carried out according to the manufacturer’s instructions. The sequencing was performed on the MinION Mk1B (MinKNOW 24.06.8 and High-accuracy model v4.3.0) and Flow Cell R10 (FLO-MIN114). The Q score threshold was six. The sequencing run was adjusted to 24 hrs.

### Data analysis

Sequencing data in Fastq format was retrieved from the device and used for data analysis. The EPI2ME Labs program was used (Oxford Nanopore Technologies, Oxford, United Kingdom), specifically the workflows metagenomics v2.11.0 and Flu v1.2.3. The strain with the highest number of hits was selected as top hit.

The fastq files were uploaded to Geneious Prime (2023.2.1). The raw data was mapped against a reference database (S1 File) using minimap2.24 (ont-sensitive data type). The strain with the highest number of hits, coverage depth and breadth was selected as the top hit. Reads mapped to the database of influenza A virus (IAV) segments were used to perform a reference mapped assembly [38]. The chosen consensus sequence contig was the one with the highest number of mapped reads. If the gene length is not fully covered, the necessary contigs are re-assembled until the whole gene is covered. Sequences identified in this study were submitted to GenBank with the accession numbers indicated in S3 Table.

### Phylogenetic analysis

For phylogenetic analysis, the obtained nucleotide sequences of the isolated AIV viruses and global and local representative HPAI H5N1 and LPAI H9N2 virus sequences were used. These were obtained from GISAID and NCBI platforms representing different geographical locations and selected according to the availability of complete gene sequences. The nucleotide sequences were aligned and the mutation analysis performed using BioEdit 7.2 software. The phylogenetic trees were constructed using the Maximum likelihood method for the eight gene segments [39]. A bootstrap of 1000 iterations of the Clustal W alignment tool [40] was used to perform the phylogenetic evaluation with the MEGA version 6 program.

## Results

### Result of Real-time RT-PCR

Thirty-two out of the 50 tested flocks were positive for either H9 and/or H5 AIV; 21 flocks were positive for H9 (three positive for H9 only, ten positive H9 + IBV, five positives for H9 + ND, and three positive for H9 + H5) while 11 flocks were positive for H5 (four positive for H5 only, three positive H5 + H9, and four positives for H5 + ND). In addition, ten flocks tested positive for NDV alone and three flocks were positive for NDV + IBV, while eight flocks were negative for all viruses (Table 2).

**Table 2:** The results of real-time RT-PCR.

Results of real-time RT-PCR	Nr. of samples
Negative	8
H9N2	3
H9N2+IBV	10
H9N2+NDV	5
H9N2+H5N1	3
H5N2	1
H5N1	3
H5N1+NDV	4
NDV	10
NDV + IBV	3
Total	50

### Virus pathogenicity

Samples from seven flocks that tested positive only for H5 (four flocks) or H9 (three flocks) AIV without detection of NDV or IBV by real-time RT-PCR were selected for isolation. The history of these flocks is shown in S1 Table. The embryos of the majority of inoculated eggs were dead between the second and the fourth day post inoculation, and showed hemorrhages and/or congestion, while the control embryo developed normally. The HA titer of allantoic fluid ranged from 3–6 log2 for H5 positive samples, while H9 positive samples showed a HA titer from 6–8 log2. Afterwards, purity was confirmed by real-time RT-PCR.

### Subtyping by whole genome sequencing

**Fig 1:**
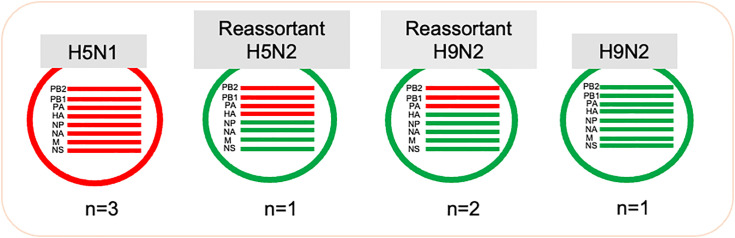
H5 and H9 Viruses reassortment of isolates used in the study. Three isolates were subtyped as HPAI H5N1 isolates and one reassortant HPAI H5N2 isolate that resulted from reassortment between H5N1 and H9N2 AI viruses, where H5N1 AIV acquired four gene segments (NP, NA, M, and NS) from H9N2 virus. The H9N2 AN2 isolate showed no evidence for reassortment by phylogenetic analysis and all eight gene segments were related to H9N2 Egyptian virus. Two reassortant LPAI H9N2 isolates were detected and resulted from reassortment between (PB1, PB2, and PA) H5N1 of clade 2.3.4.4b and H9N2 of G5.6 AI viruses.

The sequencing of the samples produced between 9.99–52.4 megabases each, leading to a calculated genome coverage of 740–3881 for IAV. The accession numbers for sequences of all gene segments of the seven chicken AIV isolates were listed in S3 Table. Phylogenetic analysis was performed for the obtained sequences for subtyping of H5 and H9 viruses. Phylogenetic analysis revealed that three isolates were subtyped as HPAI H5N1 isolates (AN1, AN7 & AN8) and one reassortant HPAI H5N2 isolate (AN6) that resulted from reassortment between H5N1 and H9N2, where H5N1 virus acquired four gene segments (NP, NA, M, and NS) from H9N2 virus (Fig 1).

**Fig 2:**
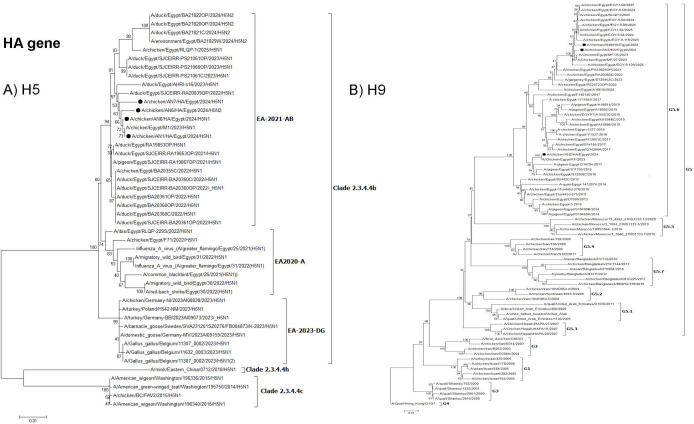
Phylogenetic tree of the HA gene: A) Egyptian H5 viruses clade 2.3.4.4b (EA-2021-AB) viruses and B) Egyptian H9N2 viruses related to G5.6. The viruses used in this study are indicated by a black dot.

The phylogenetic analysis of the HA and NA genes sequences indicated that the H5N1 isolates and the reassortant H5N2 isolate clustered with clade 2.3.4.4b (EA-2021-AB) viruses (Fig 2A). The HA and NA genes of H9N2 viruses isolated in this study related to G5.6 (Fig 2B).

The H9N2 AN2 isolate showed no evidence for reassortment by phylogenetic analysis and all eight gene segments were related to H9N2 Egyptian virus. Two reassortant LPAI H9N2 isolates (AN3 and AN4) were detected and resulted from reassortment between H5N1 of clade 2.3.4.4b (EA-2021-AB) and H9N2 of G5.6 AI viruses, where H9N2 AIV acquired the polymerase gene segments (PB1, PB2, and PA) from the HPAI H5N1 virus (Figs 4–6). This is the first described case for LPAI reassortant H9N2 that acquires gene segments from an HPAI H5N1 virus clade 2.3.4.4b (EA-2021-AB).

**Fig 3:**
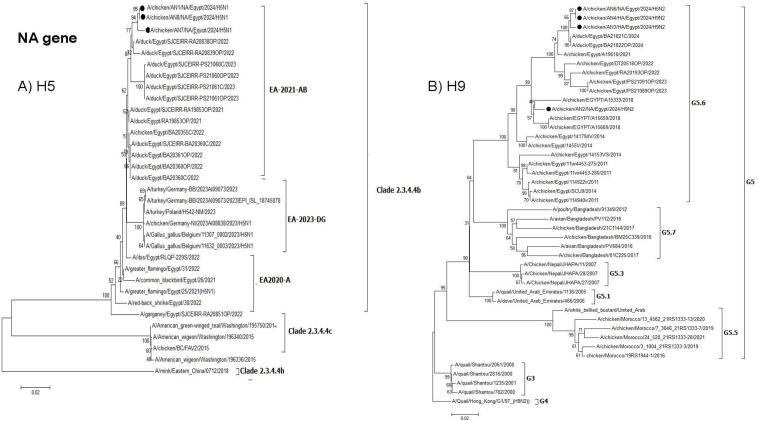
Phylogenetic tree of the NA gene: A) Egyptian H5 viruses clade 2.3.4.4b (EA-2021-AB) viruses and B) Egyptian H9N2 viruses (G5.6. lineage). The viruses used in this study are indicated by a black dot.

The AN6 isolate represents H5N2 reassortant and its NA, NP, M, and NS genes clustered within G5.6 of H9N2 viruses (Figs 3, 7, 8 and 9).

**Fig 4:**
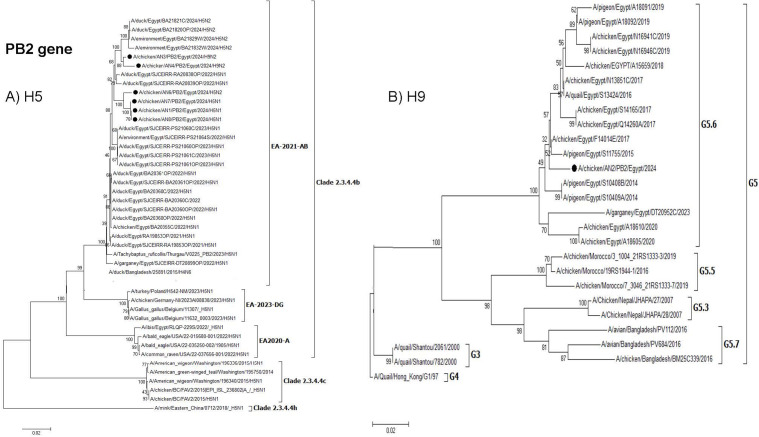
Phylogenetic tree of the PB2 gene: A) Egyptian H5 viruses clade 2.3.4.4b (EA-2021-AB) viruses and B) Egyptian H9N2 viruses (G5.6. Lineage). The viruses used in this study are indicated by a black dot.

**Fig 5:**
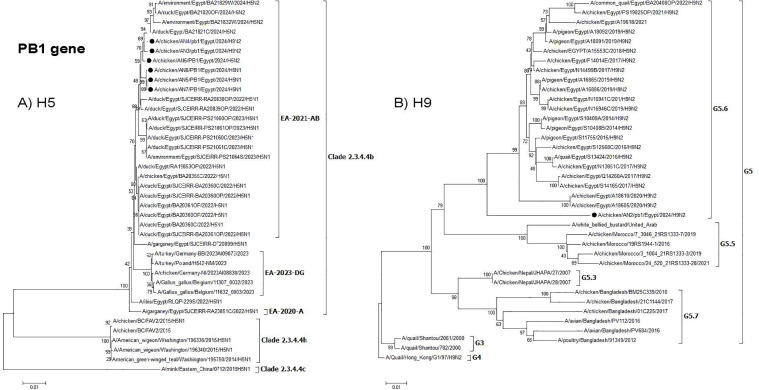
Phylogenetic tree of the PB1 gene: A) Egyptian H5 viruses clade 2.3.4.4b (EA-2021-AB) viruses and B) Egyptian H9N2 viruses (G5.6 Lineage). The viruses used in this study are indicated by a black dot.

**Fig 6:**
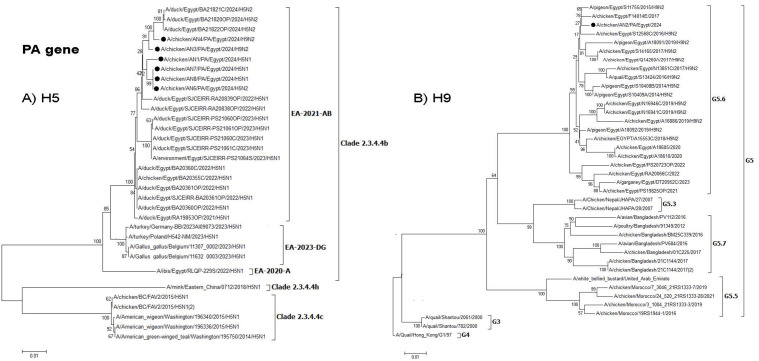
Phylogenetic tree of the PA gene: A) Egyptian H5 viruses clade 2.3.4.4b (EA-2021-AB) viruses and B) Egyptian H9N2 viruses (G5.6 Lineage). The viruses used in this study are indicated by a black dot.

**Fig 7:**
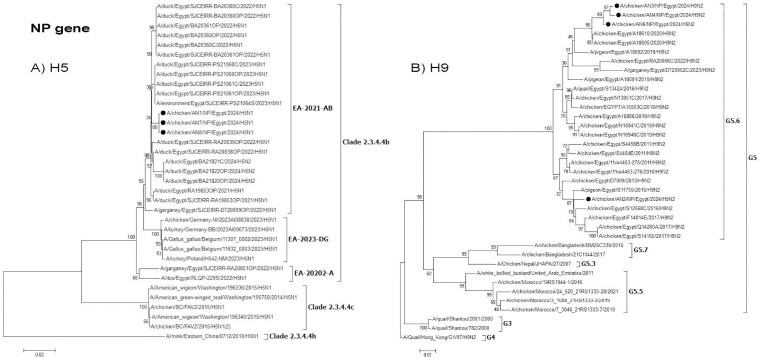
Phylogenetic tree of the NP gene: A) Egyptian H5 viruses clade 2.3.4.4b (EA-2021-AB) viruses and B) Egyptian H9N2 viruses (G5.6 Lineage). The viruses used in this study are indicated by a black dot.

**Fig 8:**
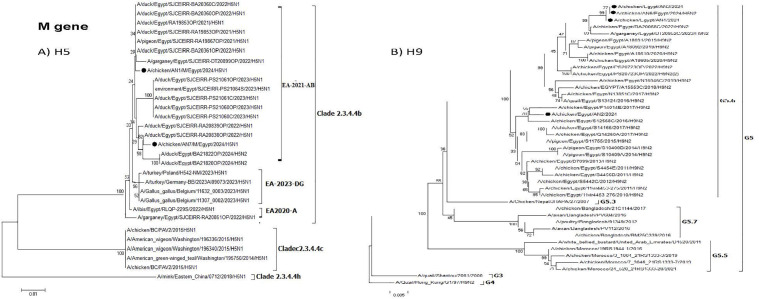
Phylogenetic tree of the M gene: A) Egyptian H5 viruses clade 2.3.4.4b (EA-2021-AB) viruses and B) Egyptian H9N2 viruses (G5.6 Lineage). The viruses used in this study are indicated by a black dot.

**Fig 9:**
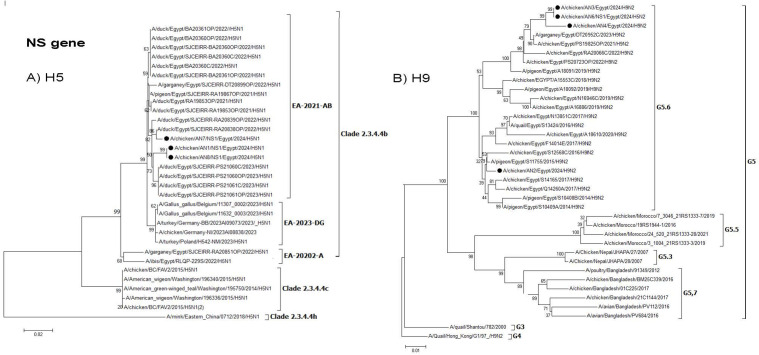
Phylogenetic tree of the NS gene: A) Egyptian H5 viruses clade 2.3.4.4b (EA-2021-AB) viruses) and B) Egyptian H9N2 viruses (G5.6 Lineage). The viruses used in this study are indicated by a black dot.

### Mutation analysis

The HA gene of the isolated HPAI viruses of H5N1 (AN1, AN7, and AN8) and H5N2 (AN6) isolate have similar multi-basic amino acids (321PLREKRRKR/GLF332) indicating HPAI viruses. No amino acid mutations in the major antigenic sites, receptor binding sites (RBS), and cleavage site between isolated H5N1/H5N2 viruses in this study and the previously isolated viruses in Egypt either from domestic or wild birds were observed (Table 3).

**Table 3:** The antigenic and cleavage sites of the HA gene of H5N1viruses from this study.

	A	B		E					
Number of nucleotides	140	141	154	156	184	71	83	86	321-332
**A/ibis/Egypt/RLQP-229S/2022**	A	P	N	A	A	I	A	A	PLREKRRKRGLF
**A/migratory wild bird/Egypt/30/2022/H5N1**	-	-	-	-	-	-	-	-	-
**Influenza A virus (A/greater flamingo/Egypt/31/2022**	-	-	-	-	-	-	-	-	
**A/duck/Egypt/SJCEIRR-BA20360OP/2022**	-	-	-	-	-	-	-		-
**A/duck/Egypt/SJCEIRR-PS21061OP/2023**	-	-	-	-	-	-	-		-
**A/chicken/Egypt/RLQP-1/2025**	-	-	-	-	-	-	-		-
**AN1/AN6/ AN7/ AN8**	-	-	-	-	-	-	-	-	-

These four viruses have Q222 and G224 at the receptor binding site and that suggested these viruses have high binding affinity to the avian-type receptor [41]. All H5 viruses have I146T, N221K as other Egyptian viruses isolates in 2022–2023. AN7 and AN8 have specific mutation (E390K) comparing with the original virus A/ibis/Egypt/RLQP-229S/2022.

The sequence of HA cleavage site of the isolated H9N2 viruses was PARSGR/GLF in AN2 isolate, while in AN3 and AN4 isolates was PARSSR/GLF, which indicates LPAI viruses [42]. The three H9N2 viruses isolated in this study had several amino acid residues in the RBS of the HA gene that are associated with a preference for binding to human-like α2,6 sialic acid including 191H, 232N, 234L, 235I, and 236G (Table 3). Moreover, they had S166N amino acid mutation in antigenic site I compared to the G1/97 ancestral strain (Table 4). The viruses had I132V, T413N, I422V as other Egyptian viruses isolated in 2024 with specific mutations at K321R in AN3 and S337G in AN2 And A451S in AN4.

**Table 4:** The receptor binding site of HA of the H9N2 viruses.

Number of nucleotides	166	191	197	198	232	234	235	236	399
A/quail/Hong Kong/G1/97	**S**	H	T	**E**	N	L	**Q**	G	K
A/chicken/AN2/HA/Egypt/2024/H9N2	N	H	T	A	N	L	I	G	K
A/chicken/AN3/HA/Egypt/2024/H9N2	N	H	T	A	N	L	I	G	K
A/chicken/AN4/HA/Egypt/2024/H9N2	N	H	T	A	N	L	I	G	K

The NA gene mutation analysis revealed that the three H5N1 viruses (AN1, AN7, and AN8) shared I19M and I396M amino acid mutations with Egyptian viruses isolated in 2024, while the V448I mutation was unique to the viruses in this study, when compared to the representative field strain A/ibis/Egypt/RLQP-229S/2022. No substitutions of amino acids related to oseltamivir antiviral resistance were found in all H5N1 isolates of this study. Also, the H9N2 isolates AN2, AN3, and AN4, plus H5N2 (AN6) isolate showed no mutations related to NA antiviral resistance. However, its sialic-acid-binding pocket of the hemadsorption site (366–373, 399–404, and 431–433) revealed mutations in several forms as shown in Table 5.

**Table 5:** The sequences reported in the Hemadsorption site for AI isolates containing the N2 gene.

Strains	Hemadsorption site		
	1^st^ loop 366-373	2^nd^ loop 399-404	3^RD^ loop 431-433
A/quail/Hong Kong/G1/97	IK**K**DSR	DS**DI**RS	PQE
A/chicken/AN2/HA/Egypt/2024/H9N2	IK**K**DSR	DS**DNWS**	PQE
A/chicken/AN3/HA/Egypt/2024/H9N2	IKTDSR	DSNDRS	PQE
A/chicken/AN4/HA/Egypt/2024/H9N2	IKTDSR	DSNDRS	PQE
A/chicken/AN6/HA/Egypt/2024/H5N2	IKTDSR	DSNDRS	PQE

Stalk length mutational analysis showed that there were no stalk deletions at sites 38–39, which are a feature of viruses that resemble G5.6 [43]. Additionally, the particular stalk deletion at amino acids 46–50 is absent, which is crucial for the viral’s adaptation in poultry [42]. The AN4 and AN6 isolates had specific mutations at T19A, I20V, and G318S. TheAN3, AN4, AN6 isolates had P79H, I254V, R268K, and N328I. Also, the AN2 strain had V106I, G125S, K143R, V156I, G246S, I20L and A50V compared to the G1/97 ancestral strain.

All isolated IAV in this study had 13P in PB1 gene and 82S in PB1-F2 gene specific for mammalian receptors preferences (S5 Table). All viruses have specific mutations at T156A, T296I in AN4, AN6, AN8, and E187V, M179V in AN3 compared to A/ibis/Egypt/RLQP-229S/2022 H5N1 virus. Also, the AN2 had L212V, T213D, S216, A374V, S633N compared to the G1/97 ancestral strain.

All viruses isolated in this study had specific mutations in PB2. The AN2, AN3 viruses had V504 as a virulent marker (S4 Table). Also, the AN4 had R427N, G477E, T491M, P515L, K571N, Q591R, Y592S, S592R and N715S. The Q591R enhanced the polymerase activity and virus replication in humans. The AN1, AN6, AN7, AN8 had V504A, R508K (that increase susceptibility to human), G509R, S514L and P515S. AN1 and AN8 have N100S which increase susceptibility to mammalian hosts and R136Q which is a cold-adapted mutation compared to A/ibis/Egypt/RLQP-229S/2022 H5N1 virus. Compared to the G1/97, the AN2 strain has specific mutations at S155N which promote mammalian adaptation, as well as M575I and K699R.

Moreover, all H5N1 and H9N2 viruses that were isolated in this study have 127V and 672L in the PA gene that act as virulence markers (S4 Table). The four H5 viruses isolated in this study have I61T, N115S (which are more prevalent in human viruses), I178V, S451C like all Egyptian H5N1 clade 2.3.4.4(EA-2021-AB), while, they have D216N and S705Y mutations unlike other Egyptian H5N1 clade 2.3.4.4(EA-2021-AB). Moreover, the AN2 had P266S, R269K, E327G, H437Y, K539R compared to the G1/97 ancestral strain.

All H5N1 and H9N2 viruses that were isolated in this study have 398Q, 28I/V in the NP and M2 gene and AN2, AN3, AN4, AN6 had 15I, 55F in M1, M2, respectively (S5 Table). Also, all viruses have 64S, 69P in M2 and 42S in NS1 and AN2, AN3, AN4 have 189D in NS1 as virulent marker gene (S4 Table). The viruses in this study have specific mutation at Q52H, V105I, I183V, M374I and T396I in both AN3, AN4 in NP gene and Q211R in AN3 in M1 gene compared with G1/97 ancestral strain. AN3 and AN4 have L10P, R12K, G16E, R18N in M2 gene, P216T in NS1 and P58T in NS2 and AN2 have V28I, I32V in M2 gene compared with G1/97 ancestral strain. Also, AN1 and AN8 have I77F, T197I, A223E in M2 gene and AN7 has R78C, I137V in M2 gene compared to A/ibis/Egypt/RLQP-229S/2022 H5N1.

## Discussion

The aim of this study was to investigate the molecular epidemiology of avian respiratory viruses circulating in Egyptian poultry flocks during 2024, with a particular focus on the co-circulating HPAI H5N1 and LPAI H9N2 viruses. The full genome sequencing for seven AIV isolates was performed with Oxford Nanopore sequencing. They represent the main co-circulating HPAI H5N1 and LPAI H9N2 viruses during 2024. Results revealed a H5N2 reassortant (AN6 isolate) resulting from reassortment between H5N1 and H9N2 AI viruses, where H5N1 AIV acquired four gene segments from H9N2 virus (NP, NA, M, and NS). Moreover, two H9N2 reassortants were detected (AN3 & AN4) as a result of reassortment between H5N1 and H9N2 AI viruses, where H9N2 AIV acquired the polymerase segments (PB1, PB2, and PA) from the HPAI H5N1 virus.

The HPAI H5N1 clade 2.3.4.4b, first detected in Europe in 2020, spread rapidly worldwide following its establishment in wild birds [44]. It was identified in Egypt during the 2021/2022 season, where it quickly replaced the previously circulating HPAI H5N8 clade 2.3.4.4b viruses [31]. Co-circulation of H5N1 with endemic H9N2 AIV in poultry increases the risk of reassortment and emergence of novel viruses, underscoring the urgent need for continuous surveillance and whole-genome sequencing.

Nanopore sequencing offers a rapid, cost-effective, and portable approach for full genome characterization of AIV. Unlike traditional sequencing methods, it allows for generation of long reads, which facilitates the accurate assembly of complete viral genomes [45,46]. This is particularly crucial in the context of co-circulating subtypes such as H5N1 and H9N2, where genome reassortment is a major concern. Through comprehensive genomic surveillance using ONT, it becomes possible to detect reassortant viruses at an early stage, monitor their evolution, and assess their potential risks to poultry and public health [46,47]. Other molecular techniques for reassortant identification include RT-PCR with segment-specific primers and restriction fragment length polymorphism (RFLP) [48]. However, whole genome sequencing has been successfully used recently, as it provides complete information about all eight gene segments and their origins [49,50]. Here, long-read sequencing such as Nanopore offer advantages, due to its portability, speed and real time analysis. It also allows a scalable throughput for many settings due to different device sizes and protocols, making local surveillance more affordable [51–53].

During 2024, respiratory disease outbreaks were widespread in both backyard and commercial broiler flocks across seven Egyptian governorates, with mortality rates ranging from 7% to 80%. More than half of the examined flocks tested positive for avian influenza viruses, with H9N2 remaining the predominant subtype, confirming its endemic circulation. Co-infections were frequently detected, particularly H9N2 with IBV or NDV, highlighting the complex, multifactorial nature of respiratory disease and the potential for synergistic interactions that exacerbate clinical outcomes. Importantly, H5-positive flocks were also identified, including mixed infections with H9N2 or NDV, which raises concerns about viral reassortment and the emergence of novel variants [54,55]. The presence of negative flocks further suggests that other infectious agents or non-infectious factors may contribute to respiratory disease. Overall, these findings emphasize the critical need for continuous molecular surveillance, especially in regions with high poultry density and frequent co-infections, where viral evolution may compromise effective control measures.

Previously, two reports for reassortment events between the H5N8 and H9N2 viruses have been reported, after almost two years of co-circulation of both AIV subtypes in Egyptian poultry. The H5N2 reassortant virus has been detected in ducks with only one gene segment (NA) acquired from H9N2 into H5N8 virus, and another H5N2 reassortant has been detected in chickens where the HPAI H5N8 virus acquired internal gene segments from the LPAI H9N2 virus [26,27]. Recently, a novel reassortant HPAI H5N2 virus was detected in ducks due to reassortment between HPAI H5N1 virus clade 2.3.3.4b (EA-2021-AB) and LPAI H9N2 virus (G5.6) [31], In this study, a H5N2 reassortant (AN6) was detected in backyard chickens in May 2024, where the H5N1 virus acquired NP, NA, M and NS gene segments from the H9N2 (G5.6) virus. These observations indicated the frequent reassortment between the endemic H5Nx clade 2.3.4.4b viruses with the co-circulating H9N2 viruses with different constellation patterns of internal genes. The ability of the newly discovered 2024/H5N2 reassortant to be established in poultry is questionable and needs continuous monitoring in further studies.

Interestingly, two H9N2 reassortants (AN3 & AN4) were detected, where the H9N2 acquired the polymerase genes (PB2, PB1, and PA) from the HPAI H5N1 clade 2.3.4.4b (EA-2021-AB) virus. The PB2, PB1, and PA genes from these H9N2 reassortant viruses were clustered with duck H5N1 viruses ( Figs 3–5), which indicated that reassortment events may be taking place in ducks as recently reported. Moreover, several amino acid mutations in the NP, M, and NS genes were detected in the two H9N2 reassortants but were not found in previous Egyptian H9N2 viruses. These mutations might be considered as adaptive mutations for the new reassortants after reassortment in ducks that were acquired during their replication in chickens. The detection of these reassortants in commercial broiler farms in two different governorates indicated that these viruses were already established in commercial chickens and acquired their fitness through these adaptive mutations. Additional studies on the pathogenicity in different bird species reared in Egypt and coinfection studies are needed. Moreover, the efficacy of commercially available H9N2 vaccines should be further investigated.

Several mutations potentially associated with increased virulence and mammalian adaptation were detected in internal genes. All H5 and H9N2 viruses under study had 13P in PB1, 82S in PB1-F2, 398Q in NP gene, 28I/V in M2,N100S in PB2 gene of AN1 and AN8 H5N1 isolates that increase susceptibility to mammalian receptors [56,57]. All H5 viruses have V504A, R508K in PB2 and N115S in PA [58,59] and Q591R in AN4, and S155N in AN2 in PB2 that increase susceptibility to human receptor preferences [60,61]. Moreover, residues V504 in PB2 gene in AN2, AN3 and 127V and 672L in PA gene, 64S and 69P in M2 gene, and 42S in NS1 gene that was present in all H5 and H9N2 viruses under study were considered as virulence markers [61–63]. Moreover, residue 189D present in all H9N2 viruses under study is also considered as a virulence marker [64]. These observations highlight the evolution of AIV in Egypt due to reassortment or point mutations which continuously happened. This potentially results in increased virulence and higher zoonotic potential of the viruses. Nonetheless, controlled challenge experiments in diverse poultry species would be needed to determine the actual magnitude of the threat.

In conclusion, this study documents the emergence of reassortant AIVs arising from co-circulating LPAI H9N2 G5.6 and HPAI H5N1 clade 2.3.4.4b (EA-2021-AB) viruses in both commercial and backyard chickens in Egypt. These findings highlight the need to assess the pathogenicity of these reassortants and the effectiveness of existing vaccines, and they emphasize the importance of continuous molecular surveillance to monitor their potential spread and mitigate potential economic losses.

## Supporting information

S1 Table The epidemiological data of clinical samples. (DOCX)

S2 Table The primers used for suspected pathogen detection by real time RT-PCR. (DOCX)

S3 Table The accession numbers (AN) for the sequences of the 8 gene segments of the 7 isolates. (DOCX)

S4 Table The virulence markers of H5 and H9 viruses. (DOCX)

S5 Table Mammalian and avian preference of amino acids mutation of H5N1 viruses. (DOCX)

S1 File The reference database used for mapping the raw data with minimap2.24 (ont-sensitive data type). (DOCX)

S2 File Graphical abstract. (PNG)
