# Supplementary material for: Concurrent circulation of avian influenza viruses H5N1 and H9N2 enhances the genetic evolution of reassortant viruses in Egyptian poultry populations
Source: PLoS One. 2026 May 8;21(5):e0348609. doi: 10.1371/journal.pone.0348609 (PMC13155612; doi:10.1371/journal.pone.0348609)
Supplement: S1 Table — (DOCX) [file pone.0348609.s001.docx]

**Supplementary 1 Table.** The epidemiological data of clinical samples.

| **Result of PCR** | **Flock Mortality** | **History of vaccination** | **Farm Type** | **species** | **Country** | **Date of collection** | **Number** |
| --- | --- | --- | --- | --- | --- | --- | --- |
| H9N2+NDV | 50% | No | commercial | chicken | Assiut | 12-5-2024 | 1 |
| H5N1+NDV | 50% | No | backyard | chicken | Gharbiya | 10-2-2024 | 2 |
| NDV | 40% | vaccinated | commercial | chicken | Kafr El Sheikh | 22-8-2024 | 3 |
| H9N2+IBV | 35% | vaccinated | commercial | chicken | Dakahlia | 1-9-2024 | 4 |
| H5N1+NDV | 60% | No | backyard | chicken | Kafr El Sheikh | 8-3-2024 | 5 |
| H9N2+IBV | 30% | No | commercial | chicken | Dakahlia | 4-8-2024 | 6 |
| NDV | 30% | No | backyard | chicken | Gharbiya | 15-3-2024 | 7 |
| H9N2+NDV | 40% | No | backyard | chicken | Assiut | 7-7-2024 | 8 |
| NDV+IBV | 45% | No | backyard | chicken | Aswan | 13-6-2024 | 9 |
| H9N2 | 30% | vaccinated | commercial | chicken | Gharbiya | 3-1-2024 | 10 |
| H5N1 | 50% | vaccinated | backyard | chicken | Assiut | 10-9-2024 | 11 |
| H9N2+NDV | 50% | vaccinated | commercial | chicken | Aswan | 12-11-2024 | 12 |
| NDV | 50% | No | commercial | chicken | Qena | 5-5-2024 | 13 |
| Neg | 10% | vaccinated | backyard | chicken | El-Wadi El-gadid | 30-8-2024 | 14 |
| H9N2+IBV | 20% | vaccinated | commercial | chicken | Kafr El Sheikh | 20-2-2024 | 15 |
| NDV | 45% | vaccinated | commercial | chicken | Assiut | 17-9-2024 | 16 |
| H9N2+NDV | 42% | No | commercial | chicken | El-Wadi El-gadid | 15-4-2024 | 17 |
| H9N2+H5N1 | 70% | No | backyard | chicken | Qena | 15-3-2024 | 18 |
| H5N1+NDV | 80% | No | backyard | chicken | Dakahlia | 21-11-2024 | 19 |
| H9N2+H5N1 | 60% | No vaccination | backyard | chicken | El-Wadi El-gadid | 10-12-2024 | 20 |
| NDV | 60% | vaccinated | commercial | chicken | Assiut | 2-10-2024 | 21 |
| H9N2+IBV | 15% | vaccinated | commercial | chicken | Dakahlia | 7-9-2024 | 22 |
| H5N1+NDV | 75% | No | backyard | chicken | El-Wadi El-gadid | 16-8-2024 | 23 |
| H9N2+NDV | 45% | vaccinated | commercial | chicken | Qena | 15-12-2024 | 24 |
| NDV+IBV | 30% | Vaccinated | commercial | chicken | Kafr El Sheikh | 3-5-2024 | 25 |
| H9N2+H5N1 | 40% | No | backyard | chicken | Assiut | 15-3-2024 | 26 |
| H9N2+IBV | 12% | vaccinated | commercial | chicken | Gharbiya | 17-8-2024 | 27 |
| NDV | 40% | vaccinated | commercial | chicken | Assiut | 24-9-2024 | 28 |
| H9N2+IBV | 30% | vaccinated | commercial | chicken | Dakahlia | 17-8-2024 | 29 |
| H5N1 | 50% | vaccinated | backyard | chicken | Qena | 5-4-2024 | 30 |
| NDV | 60% | No | backyard | chicken | Assiut | 22-3-2024 | 31 |
| H5N2 | 20% | No | backyard | chicken | Aswan | 18-1-2024 | 32 |
| H9N2 +IBV | 35% | vaccinated | commercial | chicken | Qena | 25-2-2024 | 33 |
| H9N2+IBV | 15% | No | commercial | chicken | Dakahlia | 16-2-2024 | 34 |
| NDV+IBV | 5% | No | backyard | chicken | Gharbiya | 14-3-2024 | 35 |
| H9N2+IBV | 25% | No | commercial | chicken | Assiut | 25-9-2024 | 36 |
| Neg | 20% | No | backyard | chicken | Qena | 1-11-2024 | 37 |
| NDV | 40% | vaccinated | commercial | chicken | El-Wadi El-gadid | 2-6-2024 | 38 |
| NDV | 55% | No | backyard | chicken | Assiut | 15-7-2024 | 39 |
| Neg | 25% | vaccinated | commercial | chicken | Gharbiya | 25-8-2024 | 40 |
| Neg | 20% | No | backyard | chicken | Dakahlia | 20-11-2024 | 41 |
| H9N2 | 35% | No | commercial | chicken | Aswan | 22-12-2024 | 42 |
| H5N1 | 60% | vaccinated | backyard | chicken | El-Wadi El-gadid | 18-12-2024 | 43 |
| Neg | 22% | No | backyard | chicken | Aswan | 12-1-2024 | 44 |
| Neg | 15% | No | backyard | chicken | Dakahlia | 1-3-2024 | 45 |
| H9N2+IBV | 30% | vaccinated | commercial | chicken | Gharbiya | 12-1-2024 | 46 |
| Neg | 22% | No | backyard | chicken | Dakahlia | 14-5-2024 | 47 |
| Neg | 22% | Vaccinated | commercial | chicken | Assiut | 27-4-2024 | 48 |
| H9N2 | 16% | vaccinated | commercial | chicken | Kafr El Sheikh | 12-11-2024 | 49 |
| NDV | 40% | No | backyard | chicken | El-Wadi El-gadid | 7-8-2024 | 50 |
