# Supplementary material for: Concurrent circulation of avian influenza viruses H5N1 and H9N2 enhances the genetic evolution of reassortant viruses in Egyptian poultry populations
Source: PLoS One. 2026 May 8;21(5):e0348609. doi: 10.1371/journal.pone.0348609 (PMC13155612; doi:10.1371/journal.pone.0348609)
Supplement: S2 Table — (DOCX) [file pone.0348609.s002.docx]

**Supplementary 2 Table.** The primers used for suspected pathogen detection by real time RT-PCR.

| **GENE** |  | **Sequence** | **REF** |
| --- | --- | --- | --- |
| IBV | AIBV-F | ATGCTCAACCTTGTCCCTAGCA | Meir et al., 2010 |
|  | AIBV-R | TCAA-ACTGCGGATCA-TCACGT |  |
|  | AIBV probe | FAM-TTGGAAGTAGAGTGACGCC-CAAACTTCA-BHQ1 |  |
| NDV | NDV-F | TCCGGAGGATACAAGGGTCT | Wise et al., 2004 |
|  | NDV-R | AGCTGTTGCAACCCCAAG |  |
|  | NDV-probe | FAM-AAGCGTTTCTGTCTCCTTCCTCCA-TAMRA |  |
| H5 | H5LH1 | ACATATGACTAC CCACARTATTCA G | Lȍndt et al., 2008 |
|  | H5RH1 | AGACCAGCT AYC ATGATTGC |  |
|  | H5PRO | FAM-TCWACA GTGGCGAGT TCCCTAGCA-AMRA |  |
| H9 | H9F | GGAAGAATTAATTATTATTGGTCGGTAC | Ben Shabat et al., 2010 |
|  | H9R | GCCACCTTTTTCAGTCTGACATT |  |
|  | H9 Probe | FAM-AACCAGGCCAGACATTGCGAGTAA GATCC-TAMRA |  |
| H7 | H7-For | \|  \| TTTGGTTTAGCTTCGGG \| \| --- \| --- \| | Lȍndt et al., 2008 |
|  | H7-deg Rev | \|  \| GAAGAA(C)AAGGCC(T)CATTG \| \| --- \| --- \| |  |
|  | H7 probe | \|  \| ROX-CATCATGTTTCATACTTCTGGCCAT-TAMRA \| \| --- \| --- \| |  |
| N1 | N1 forward | TAYAACTCAAGGTTTGAGTCTGTYGCTTG | Li et al., 2013 |
|  | N1 reverse | ATGTTRTTCCTCCAACTCTTGATRGTGTC |  |
|  | N1 Probe | FAM-TCAGCRAGTGCYTGCCATGATGGCA-TAMRA |  |
| N2 | N2-F | AGT CTG GTG GAC YTC AAA YAG | Hoffmann et al., 2016 |
|  | N2-R | AAT TGC GAA AGC TTA TAT AGV CAT |  |
|  | IN2-probe | FAM-CCA TCA GGC CAT GAG CCT-Tamra |  |
| N8 | N8-1296F | TCC ATG YTT TTG GGT TGA RAT GAT | Hoffmann et al., 2016 |
|  | N8-1423R | GCT CCA TCR TGC CAY GAC CA |  |
|  | N8-probe | FAM- TCH AGY AGC TCC ATT GTR ATG TGT GGA GT-TAMRA |  |
