# Supplementary material for: Concurrent circulation of avian influenza viruses H5N1 and H9N2 enhances the genetic evolution of reassortant viruses in Egyptian poultry populations
Source: PLoS One. 2026 May 8;21(5):e0348609. doi: 10.1371/journal.pone.0348609 (PMC13155612; doi:10.1371/journal.pone.0348609)
Supplement: S3 Table — (DOCX) [file pone.0348609.s003.docx]

**Supplementary 3 Table.** The accession numbers (AN) for the sequences of the 8 gene segments of the 7 isolates.

| **Name** | **HA** | **NA** | **PA** | **PB1** | **PB2** | **NP** | **M1** | **M2** | **NS1** | **NS2** |
| --- | --- | --- | --- | --- | --- | --- | --- | --- | --- | --- |
| AN1 | PV554207 | PV554277 | PV569560 | PV577919 | PV569573 | PV569609 | PV571668 | PV569899 | PV571665 | PV569895 |
| AN2 | PV579976 | PV580077 | PV580247 | PV582993 | PV588939 | PV589092 | PV589536 | PV589115 | PV589539 | PV589283 |
| AN3 | PV579977 | PV580078 | PV580248 | PV582994 | PV588940 | PV589093 | PV589537 | PV589116 | Pv589540 | PV589284 |
| AN4 | PV579978 | PV580079 | PV580249 | PV582995 | PV588941 | PV589094 | PV589538 | PV589117 | Pv589541 | PV589285 |
| AN6 | PV590030 | PV590061 | PV590119 | PV590111 | PV590115 | PV590063 | PV590131 | PV5900103 | PV590130 | PV590071 |
| AN7 | PV554208 | PV554278 | PV569561 | PV569568 | PV569574 | PV569610 | PV571669 | PV569900 | PV571666 | PV569896 |
| AN8 | PV554209 | PV554279 | PV569562 | PV569569 | PV569575 | PV569611 | PV571670 | PV569901 | PV571667 | PV569897 |
