# Supplementary material for: Concurrent circulation of avian influenza viruses H5N1 and H9N2 enhances the genetic evolution of reassortant viruses in Egyptian poultry populations
Source: PLoS One. 2026 May 8;21(5):e0348609. doi: 10.1371/journal.pone.0348609 (PMC13155612; doi:10.1371/journal.pone.0348609)
Supplement: S4 Table — (DOCX) [file pone.0348609.s004.docx]

**Supplementary 4 Table.** The virulence markers of H5 and H9 viruses.

| **References** | **H5N2** | **H9N2** | | | **H5N1** | | | **Avirulent** | **Virulent** | **Number of aminoacids** | **Protein** |
| --- | --- | --- | --- | --- | --- | --- | --- | --- | --- | --- | --- |
|  | **AN6** | **AN4** | **AN3** | **AN2** | **AN8** | **AN7** | **AN1** |  |  |  |  |
| Wang et al., 2012 | E | E | E | E | E | E | E | E | K | 627 | PB2 |
|  | I | I | I | I | I | I | I | M/I | L | 147 |  |
|  | V | V | V | V | V | V | V | V | G | 250 |  |
| Rolling et al., 2009 | A | V | V | V | A | A | A | I | V | 504 |  |
| Teng et al., 2013 | D | D | D | D | D | D | D | D | N | 701 |  |
| Mok et al., 2014 | Q | Q | Q | Q | Q | Q | Q | Q | K | 591 |  |
| Chen et al., 2006; Lee et al., 2007 | M | M | M | M | M | M | M | M/V | I | 317 | PB1 |
| Lycett et al., 2009 | V | V | V | V | V | V | V | I | V | 127 | PA |
| Li et al., 2005 | L | L | L | L | L | L | L | F | L | 672 |  |
| Otte et al., 2015 | V | V | V | V | V | V | V | V | R | 100 |  |
| Rolling et al., 2009 | I | I | I | I | I | I | I | I | L | 550 |  |
| Chen et al., 2017 | K | K | K | K | K | K | K | K | R | 470 | NP |
| Lycett et al., 2009 | S | S | S | S | S | S | S | p | S/A/F | 64 | M2 |
| Lycett et al., 2009 | P | P | P | P | P | P | P | L | P | 69 |  |
| Jiao et al., 2008 | S | S | S | S | S | S | S | A/P | S | 42 | NS1 |
| Lee et al., 2007 | D | D | D | D | D | D | D | D | E | 92 |  |
| Dankar et al., 2011 | F | F | F | F | F | F | F | F | L | 103 |  |
|  | M | M | M | M | M | M | M | M | I | 106 |  |
| Subbarao et al., 2000 | D | D | D | D | N | N | N | N | N | 189 |  |
|  | I | I | I | I | M | M | M | M | I | 31 | NS2 |
|  | H | H | H | H | H | H | H | H/L | Y | 56 |  |
