# Supplementary material for: Concurrent circulation of avian influenza viruses H5N1 and H9N2 enhances the genetic evolution of reassortant viruses in Egyptian poultry populations
Source: PLoS One. 2026 May 8;21(5):e0348609. doi: 10.1371/journal.pone.0348609 (PMC13155612; doi:10.1371/journal.pone.0348609)
Supplement: S5 Table — (DOCX) [file pone.0348609.s005.docx]

**Supplementary 5 Table.** Mammalian and avian preference of amino acids mutation of H5N1 viruses.

| **References** | **H5N2** | **H9N2** | | | **H5N1** | | | **Mammalian**  **preference** | **Avian preference** | **Number of A.A.** | **Protein** |
| --- | --- | --- | --- | --- | --- | --- | --- | --- | --- | --- | --- |
|  | **AN6** | **AN4** | **AN3** | **AN2** | **AN8** | **AN7** | **AN1** |  |  |  |  |
| Shaw et al., 2002; Chen et al., 2006 | A | A | A | A | A | A | A | S | A | 44 | PB2 |
| Guilligay et al., 2008 | M | M | M | M | M | M | M | T | M | 64 |  |
| Shaw et al , 2002 | T | T | T | T | T | T | T | M | T | 81 |  |
| Shaw et al., 2002; Chen et al., 2006 | E | E | E | E | E | E | E | T | A | 558 |  |
| Mok et al., 2014 | Q | Q | Q | Q | Q | Q | Q | K | Q | 591 |  |
| Wang et al., 2012 | E | E | E | E | E | E | E | K | E | 627 |  |
| Kuzuhara et al., 2009 | A | A | A | A | A | A | A | T | A | 661 |  |
| Liu et al., 2013; Koçer et al., 2014 | V | V | V | V | V | V | V | I | V | 667 |  |
| Teng et al., 2013 | D | D | D | D | D | D | D | N | D | 701 |  |
| Kuzuhara et al., 2009 | K | K | K | K | K | K | K | R | K | 702 |  |
| Liu et al., 2013 | P | P | P | P | P | P | P | P | L | 13 | PB1 |
| Taubenberger et al., 2005 | V | V | V | V | V | V | V | I | V | 336 |  |
|  | N | N | N | N | N | N | N | S | N | 375 |  |
| Liu et al., 2013 | K | K | K | K | K | K | K | R | K | 73 | PB1-F2 |
| Alymova et al., 2011; Liu et al., 2013 | R | R | R | R | R | R | R | Q | R | 79 |  |
|  | S | S | S | S | S | S | S | S | L | 82 |  |
| Wanitchang et al., 2011 | P | P | P | P | P | P | P | L | P | 28 | PA |
| Shaw et al., 2002; Chen et al., 2006 | D | D | D | D | D | D | D | N | D | 55 |  |
| Chen et al., 2006 | R | R | R | R | R | R | R | Q | R | 57 |  |
| Wang et al., 2015 | V | V | V | V | V | V | V | A | V | 100 |  |
| Brown et al., 2001 | E | E | E | E | E | E | E | G | E | 133 |  |
| Finkelstein et al., 2007 | S | S | S | S | S | S | S | C | S | 225 |  |
| Yamaji et al., 2015 | C | C | C | C | C | C | C | Y | C | 241 |  |
| Finkelstein et al., 2007 | L | L | L | L | L | L | L | I | L | 268 |  |
| Chen et al., 2006 | K | K | K | K | K | K | K | R | K | 356 |  |
| Finkelstein et al., 2007 | E | E | E | E | E | E | E | D | E | 382 |  |
| Chen et al., 2006 | A | A | A | A | A | A | A | S | A | 404 |  |
| Shaw et al., 2002; Chen et al., 2006 | S | S | S | S | S | S | S | N | S | 409 |  |
| Finkelstein et al., 2007 | T | T | T | T | T | T | T | S | T | 552 |  |
| Gabriel et al., 2005 | K | K | K | K | K | K | K | L | K | 615 |  |
| Lipatov et al., 2008 | V | V | V | V | V | V | V | I | V | 33 | NP |
| Katz et al., 2000 | G | G | G | G | G | G | G | D | G | 16 |  |
| Finkelstein et al., 2007 | I | I | I | I | I | I | I | L | I | 61 |  |
| Chen et al., 2006 | I | I | I | I | I | I | I | V | I | 109 |  |
| Shaw et al., 2002 | L | L | L | L | L | L | L | M | L | 136 |  |
| Shaw et al., 2002; Chen et al., 2006 | K | K | K | K | R | R | R | K | R | 214 |  |
|  | F | F | F | F | F | F | F | Y | F | 313 |  |
| Chen et al., 2006 | Q | Q | Q | Q | Q | Q | Q | K | Q | 357 |  |
|  | E | E | E | E | E | E | E | D | E | 372 |  |
|  | Q | Q | Q | Q | Q | Q | Q | Q | K | 398 |  |
|  | D | D | D | D | D | D | D | E | D | 455 |  |
| Katz et al., 2000 | I | I | I | I | V | V | V | I | V | 15 | M1 |
| Finkelstein et al., 2007 | V | V | V | V | V | V | V | I | V | 115 |  |
| Katz et al., 2000; Finkelstein et al., 2007 | T | T | T | T | T | T | T | A | T | 121 |  |
| Shaw et al., 2002 | T | T | T | T | T | T | T | A | T | 137 |  |
| Chen et al., 2006 | T | T | T | T | T | T | T | I | T | 11 | M2 |
| Shaw et al., 2002 | E | E | E | E | E | E | E | G/D | E | 16 |  |
| Shaw et al., 2002; Chen et al., 2006 | S | S | S | S | S | S | S | S | S | 20 |  |
| Shaw et al., 2002 | V | V | V | I | I | I | I | I/V | I | 28 |  |
| Chen et al., 2006 | Y | Y | Y | Y | Y | Y | Y | Y | Y | 57 |  |
| Pan et al., 2009 | F | F | F | F | L | L | L | F | L | 55 |  |
| Liu et al., 2013 | V | V | V | V | V | V | V | A | V | 86 |  |
| Soubies et al., 2010 | E | E | E | E | E | E | E | K/R | E | 227 | NS1 |
| Liu et al., 2013; Koçer et al., 2014 |  |  |  |  | G | G | G | G | S | 70 | NEP |
