# Supplementary material for: Concurrent circulation of avian influenza viruses H5N1 and H9N2 enhances the genetic evolution of reassortant viruses in Egyptian poultry populations
Source: PLoS One. 2026 May 8;21(5):e0348609. doi: 10.1371/journal.pone.0348609 (PMC13155612; doi:10.1371/journal.pone.0348609)
Supplement: S1 File — (DOCX) [file pone.0348609.s006.docx]

**Supplementary 1. File. The reference database used for mapping the raw data with minimap2.24 (ont-sensitive data type).**

**A_seg1_PB2**

AGCAAAAGCA GGTCAATTAT ATTCAGTATG GAAAGAATAA AAGAACTACG GAATCTGATG 60

TCGCAGTCTC GCACTCGCGA GATACTGACA AAAACCACAG TGGACCATAT GGCCATAATT 120

AAGAAGTACA CATCGGGGAG ACAGGAAAAG AACCCGTCAC TTAGGATGAA ATGGATGATG 180

GCAATGAAAT ACCCAATCAC TGCTGACAAA AGGATAACAG AAATGGTTCC GGAAAGAAAT 240

GAACAAGGAC AAACTCTATG GAGCAAAATG AGTGATGCTG GATCAGATCG AGTGATGGTA 300

TCACCTTTGG CTGTAACATG GTGGAATAGA AATGGACCCG TGACAAGTAC GGTCCATTAC 360

CCAAAAGTAT ACAAGACTTA TTTTGACAAA GTCGAAAGGT TAAAACATGG AACCTTTGGC 420

CCTGTTCATT TTAGAAATCA AGTCAAGATA CGCAGAAGAG TAGACATAAA CCCTGGTCAT 480

GCAGACCTCA GTGCCAAAGA GGCACAAGAT GTAATTATGG AAGTTGTTTT TCCCAATGAA 540

GTGGGAGCCA GGATACTAAC ATCAGAATCG CAATTAACAA TAACTAAAGA GAAAAAAGAA 600

GAACTCCGAG ATTGCAAAAT TTCTCCCTTG ATGGTTGCAT ACATGTTAGA GAGAGAACTT 660

GTACGAAAAA CAAGATTTCT CCCAGTTGCT GGCGGAACAA GCAGTATATA CATTGAAGTT 720

TTACATTTGA CTCAAGGAAC GTGTTGGGAA CAAATGTACA CTCCAGGTGG AGAAGTAAGG 780

AATGACGATG TTGACCAAAG CCTAATTATT GCGGCCAGGA ACATAGTAAG AAGAGCCGCA 840

GTATCAGCAG ATCCACTAGC ATCTTTATTG GAGATGTGCC ACAGCACACA AATTGGCGGG 900

ACAAGGATGG TGGACATTCT TAGACAGAAC CCGACTGAAG AACAAGCTGT GGATATATGC 960

AAGGCTGCAA TGGGATTGAG AATCAGCTCA TCCTTCAGCT TTGGTGGGTT TACATTTAAA 1020

AGAACAAGCG GGTCATCAGT CAAAAAAGAG GAAGAAGTGC TTACAGGCAA TCTCCAAACA 1080

TTGAAGATAA GAGTACATGA GGGGTATGAG GAGTTCACAA TGGTGGGGAA AAGAGCAACA 1140

GCTATACTAA GAAAAGCAAC CAGAAGATTG GTTCAGCTCA TAGTGAGTGG AAGAGACGAA 1200

CAGTCAATAG CCGAAGCAAT AATCGTGGCC ATGGTGTTTT CACAAGAGGA TTGCATGATA 1260

AAAGCAGTTA GAGGTGACCT GAATTTCGTC AACAGAGCAA ATCAGCGGTT GAACCCCATG 1320

CATCAGCTTT TAAGGCATTT TCAGAAAGAT GCGAAAGTGC TTTTTCAAAA TTGGGGAATT 1380

GAACACATCG ACAGTGTGAT GGGAATGATT GGAGTATTAC CAGATATGAC TCCAAGCACA 1440

GAGATGTCAA TGAGAGGAAT AAGAGTCAGC AAAATGGGTG TGGATGAATA CTCCAGTACA 1500

GAGAGGGTGG TGGTTAGCAT TGATCGGTTT TTGAGAGTTC GAGACCAACG TGGGAATGTA 1560

TTATTATCTC CTGAGGAGGT CAGTGAAACA CAGGGAACTG AGAGACTGAC AATAACTTAT 1620

TCATCGTCGA TGATGTGGGA GATTAACGGT CCTGAGTCGG TTTTGGTCAA TACCTATCAA 1680

TGGATCATCA GAAATTGGGA AGCTGTCAAA ATTCAATGGT CTCAGAATCC TGCAATGTTG 1740

TACAACAAAA TGGAATTTGA ACCATTTCAA TCTTTAGTCC CCAAGGCCAT TAGAAGCCAA 1800

TACAGTGGGT TTGTCAGAAC TCTATTCCAA CAAATGAGAG ACGTACTTGG GACATTTGAC 1860

ACCACCCAGA TAATAAAGCT TCTCCCTTTT GCAGCCGCTC CACCAAAGCA AAGCAGAATG 1920

CAGTTCTCTT CACTGACTGT AAATGTGAGG GGATCAGGGA TGAGAATACT TGTAAGGGGC 1980

AATTCTCCTG TATTCAACTA CAACAAGACC ACTAAAAGAC TAACAATTCT CGGAAAAGAT 2040

GCCGGCACTT TAATTGAAGA CCCAGATGAA AGCACATCCG GAGTGGAGTC CGCCGTCTTG 2100

AGAGGGTTTC TCATTATAGG TAAGGAAGAC AGAAGATACG GACCAGCATT AAGCATCAAT 2160

GAACTGAGTA ACCTTGCAAA AGGGGAAAAG GCTAATGTGC TAATCGGGCA AGGAGACGTG 2220

GTGTTGGTAA TGAAACGAAA ACGGGACTCT AGCATACTTA CTGACAGCCA GACAGCGACC 2280

AAAAGAATTC GGATGGCCAT CAATTAATGT TGAATAGTTT AAAAACGACC TTGTTTCTAC 2340

T 2341

**A_seg2_PB1**

AGCAAAAGCA GGCAAACCAT TTGAATGGAT GTCAATCCGA CTCTACTGTT CCTAAAGGTT 60

CCAGCGCAAA ATGCCATAAG CACCACATTC CCTTATACTG GGGATCCTCC ATACAGCCAT 120

GGAACAGGAA CAGGGTACAC CATGGACACA GTCAACAGAA CACACCAATA TTCAGAGAAG 180

GGGAAGTGGA CGACAAATAC AGAAACTGGG GCACCCCAAC TCAACCCAAT TGATGGACCA 240

CTACCTGAGG ATAATGAGCC AAGTGGATAT GCACAAACAG ACTGTGTCCT GGAGGCTATG 300

GCCTTCCTTG AAGAATCCCA CCCAGGTATC TTTGAGAACT CATGCCTTGA AACAATGGAA 360

GTCGTTCAAC AAACAAGGGT GGACAAACTA ACTCAAGGTC GCCAGACTTA TGATTGGACA 420

TTAAACAGAA ATCAACCAGC AGCAACTGCA TTAGCCAACA CCATAGAAGT TTTTAGATCG 480

AATGGACTAA CAGCTAATGA ATCAGGAAGG CTAATAGATT TCCTCAAGGA TGTGATGGAA 540

TCAATGGATA AAGAGGAAAT GGAGATAACA ACACACTTTC AAAGAAAAAG GAGAGTAAGA 600

GACAACATGA CCAAGAAAAT GGTCACACAA AGAACAATAG GGAAGAAAAA ACAAAGAGTG 660

GATAAGAGAG GCTATCTAAT AAGAGCTTTG ACATTGAACA CGATGACCAA AGATGCAGAG 720

AGAGGTAAAT TAAAAAGAAG GGCTATTGCA ACACCCGGGA TGCAAATTAG AGGGTTCGTG 780

TACTTCGTTG AAACTTTAGC TAGAAGCATT TGCGAAAAGC TTGAACAGTC TGGACTCCCG 840

GTTGGGGGTA ATGAAAAGAA GGCCAAACTG GCAAATGTTG TGAGAAAAAT GATGACTAAT 900

TCACAAGACA CTGAGCTTTC TTTCACAATC ACTGGGGACA ACACTAAGTG GAATGAAAAT 960

CAAAACCCTC GAATGTTTTT GGCGATGATT ACATATATCA CAAAAAATCA ACCTGAGTGG 1020

TTCAGAAACA TCCTGAGCAT CGCACCAATA ATGTTCTCAA ACAAAATGGC AAGACTAGGA 1080

AAAGGATACA TGTTCGAGAG TAAGAGGATG AAGCTCCGAA CACAAATACC CGCAGAAATG 1140

CTAGCAAGCA TTGACCTGAA GTATTTCAAT GAATCAACAA GGAAGAAAAT TGAGAAAATA 1200

AGGCCTCTTC TAATAGATGG CACAGCATCA TTGAGCCCTG GGATGATGAT GGGCATGTTC 1260

AACATGCTAA GTACGGTTTT AGGAGTCTCG ATACTGAATC TTGGGCAAAA GAAATACACC 1320

AAGACAACAT ACTGGTGGGA TGGGCTCCAA TCCTCCGACG ATTTTGCCCT CATAGTGAAT 1380

GCACCAAATC ATGAGGGAAT ACAAGCAGGA GTGGATAGAT TCTACAGGAC CTGCAAGTTA 1440

GTGGGAATCA ACATGAGCAA AAAGAAGTCC TATATAAATA AAACAGGGAC ATTTGAATTC 1500

ACAAGCTTTT TTTATCGATA TGGATTTGTG GCTAATTTTA GCATGGAGCT TCCCAGTTTT 1560

GGAGTGTCTG GAATAAACGA GTCAGCTGAT ATGAGCATTG GAGTAACAGT GATAAAGAAC 1620

AACATGATAA ACAATGACCT TGGACCAGCA ACAGCCCAGA TGGCTCTCCA ATTGTTCATC 1680

AAAGACTACA GATATACATA TAGGTGCCAT AGAGGAGACA CACAAATTCA GACGAGAAGA 1740

TCATTCGAGC TAAAGAAGCT GTGGGATCAA ACCCAATCAA GGGCAGGACT ATTGGTATCA 1800

GATGGGGGAC CAAACTTATA CAATATCCGG AACCTTCACA TCCCTGAAGT CTGCTTAAAG 1860

TGGGAGCTAA TGGATGAGAA TTATCAGGGA AGACTTTGTA ACCCCCTGAA TCCCTTTGTC 1920

AGCCATAAAG AAATTGAGTC TGTAAACAAT GCTGTAGTGA TGCCAGCCCA TGGTCCAGCC 1980

AAAAGTATGG AATATGATGC CGTTGCAACT ACACACTCCT GGATTCCCAA GAGGAACCGC 2040

TCTATTCTCA ACACAAGCCA AAGGGGAATT CTTGAGGATG AACAGATGTA CCAAAAGTGC 2100

TGCAACTTGT TTGAGAAATT TTTCCCTAGT AGTTCATATA GGAGACCGAT TGGAATTTCT 2160

AGCATGGTGG AGGCCATGGT GTCTAGGGCC CGGATTGATG CCAGAATTGA CTTCGAGTCT 2220

GGACGGATTA AGAAGGAAGA GTTCTCTGAG ATCATGAAGA TCTGTTCCAC CATTGAAGAA 2280

CTCAGACGGC AAAAATAATG AATTTAGCTT GTCCTTCATG AAAAAATGCC TTGTTTCTAC 2340

T 2341

**A_seg3_PA**

AGCAAAAGCA GGTACTGATT CGAAATGGAA GATTTTGTGC GACAATGCTT CAACCCGATG 60

ATTGTCGAAC TTGCAGAAAA AACAATGAAA GAGTATGGAG AGGATCTGAA AATTGAAACA 120

AACAAATTTG CAGCAATATG CACCCACTTG GAGGTATGTT TCATGTATTC AGATTTTCAT 180

TTCATCAATG AACAAGGCGA ATCAATAATG GTAGAACTTG ATGATCCAAA TGCACTGTTA 240

AAGCACAGAT TCGAAATAAT CGAGGGGAGA GACAGAACAA TGGCCTGGAC AGTAGTAAAC 300

AGTATCTGCA ACACTACTGG AGCTGAAAAA CCGAAGTTTC TACCAGATTT GTATGATTAC 360

AAGGAGAACA GATTCATCGA AATTGGAGTG ACAAGAAGAG AAGTCCACAT ATATTACCTT 420

GAAAAGGCCA CTAAAATTAA ATCTGAGAAC ACACACATTC ACATTTTCTC ATTCACTGGG 480

GAGGAAATGG CCACAAAGGC AGACTACACT CTCGACGAGG AAAGCAGGGC TAGGATTAAA 540

ACCAGGCTAT TTACCATAAG ACAAGAAATG GCCAACAGAG GCCTCTGGGA TTCCTTTCGT 600

CAGTCCGAAA GAGGCGAAGA AACAATTGAA GAAAAATTTG AAATCTCAGG AACTATGCGT 660

AGGCTTGCCG ACCAAAGTCT CCCACCGAAC TTCTCCTGCC TTGAGAATTT TAGAGCCTAT 720

GTGGATGGAT TCGAACCGAA CGGCTGCATT GAGGGCAAGC TTTCTCAAAT GTCCAAAGAA 780

GTGAATGCCA AAATTGAACC TTTTCTGAAG ACAACACCAA GACCAATCAA ACTTCCTAAT 840

GGACCTCCTT GTTATCAGCG GTCCAAATTC CTCCTGATGG ATGCTTTGAA ATTGAGCATT 900

GAAGACCCAA GTCACGAAGG AGAAGGGATT CCATTATATG ATGCGATCAA GTGCATAAAA 960

ACATTCTTTG GATGGAAAGA ACCTTATATA GTCAAACCAC ACGAAAAGGG AATAAATTCA 1020

AATTACCTGC TGTCATGGAA GCAAGTATTG TCAGAATTGC AGGACATTGA AAATGAGGAG 1080

AAGATCCCAA GGACTAAAAA CATGAAGAAA ACGAGTCAAC TAAAGTGGGC TCTTGGTGAA 1140

AACATGGCAC CAGAGAAAGT AGACTTTGAC AACTGCAGAG ACATAAGCGA TTTGAAGCAA 1200

TATGATAGTG ACGAACCTGA ATTAAGGTCA CTTTCAAGCT GGATACAGAA TGAGTTCAAC 1260

AAGGCCTGCG AGCTAACTGA TTCAATCTGG ATAGAGCTCG ATGAAATTGG AGAGGACGTA 1320

GCCCCAATTG AGTACATTGC AAGCATGAGG AGGAATTATT TCACAGCAGA GGTGTCCCAT 1380

TGTAGAGCCA CTGAGTACAT AATGAAGGGG GTATACATTA ATACTGCCCT GCTCAATGCA 1440

TCCTGTGCAG CAATGGACGA TTTTCAATTA ATCCCCATGA TAAGCAAGTG CAGAACTAAA 1500

GAGGGAAGGC GAAAAACCAA TTTATATGGA TTCATCATAA AGGGAAGATC TCATTTAAGG 1560

AATGACACAG ATGTGGTAAA CTTTGTGAGC ATGGAATTTT CTCTCACTGA CCCGAGACTA 1620

GAGCCACATA AATGGGAGAA ATACTGTGTC CTTGAGATAG GAGATATGTT ACTAAGAAGT 1680

GCCATAGGCC AAATTTCAAG GCCTATGTTC TTGTATGTTA GGACAAACGG AACATCAAAG 1740

GTCAAAATGA AATGGGGAAT GGAGATGAGA CGTTGCCTCC TTCAGTCACT CCAGCAGATC 1800

GAGAGCATGA TTGAAGCCGA GTCCTCGATT AAAGAGAAAG ACATGACCAA AGAGTTTTTT 1860

GAGAATAAAT CAGAAGCGTG GCCCATTGGG GAGTCCCCCA AGGGAGTGGA AGAAGGTTCC 1920

ATTGGGAAAG TCTGTAGGAC TCTATTGGCT AAGTCAGTGT TCAATAGCCT GTATGCATCA 1980

CCACAATTGG AAGGATTTTC AGCGGAGTCA AGAAAACTGC TTCTTGTTGT TCAGGCTCTT 2040

AGGGACAACC TCGAACCTGG GACCTTTGAT CTCGGGGGGC TATATGAAGC AATTGAGGAG 2100

TGCCTGATTA ATGATCCCTG GGTTTTGCTC AATGCATCTT GGTTCAACTC CTTCCTGACA 2160

CATGCATTAA AATAGTTATG GCAGTGCTAC TATTTGTTAT CCGTACTGTC CAAAAAAGTA 2220

CCTTGTTTCT ACT 2233

**A_seg4_HA1**

AGCAAAAGCA GGGGAAAATA AAAACAACCA AAATGAAAGC AAAACTACTA GTTCTGTTGT 60

GTGCATTTAC AGCTACATAT GCAGACACAA TATGTATAGG CTACCATGCG AACAACTCAA 120

CTGACACTGT TGACACAGTA CTTGAGAAGA ACGTGACAGT GACACACTCT GTCAACCTAC 180

TTGAGGACAG TCACAACGGA AAACTATGCC GACTAAAAGG AACAGCCCCA CTACAATTGG 240

GTAATTGCAG CATTGCCGGA TGGATCTTAG GAAATCCAGA ATGCGAATCA CTGTTTTCTA 300

AGGAATCATG GTCTTACATT GCAGAAACAC CAAACCCTAA AAATGGAACA TGTTACCCAG 360

GGTATTTCGC CGACTATGAG GAACTGAGGG AGCAATTGAG CTCAGTATCA TCATTCGAGA 420

GATTTGAAAT ATTCCCCAAG GATAGCTCAT GGCCCAACCA CACTGTAACC AAAGGAGTGA 480

CGGCATCATG CTCCCATAAT GGGAAAAGCA GCTTTTACAA AAATTTGCTA TGGCTGACGG 540

AGAAGAATGG CTTGTACCCA AATCTGAGCA AGTCCTATGT AAACAAAAAG GGAAAAGAAG 600

TCCTTGTGCT ATGGGGTGTT CATCACCCGT CTAACATGGG GGACCAACGG GCCATCTATC 660

ATAAAGAAAA TGCTTATGTT TCTGTGTTGT CTTCACATTA TAGCAGAAGA TTCACCCCAG 720

AAATAGCAAA AAGACCAAAA GTAAGAGATC AAGAAGGGAG AATTAACTAC TACTGGACTC 780

TGCTGGAACC CGGGGACACA ATAATATTTG AGGCAAATGG AAATCTAATA GCGCCGTGGT 840

ACGCTTTCGC ACTGAGTAGA GGCTTTGGGT CAGGAATCAT CATCTCAAAC GCATCAATGG 900

GTGAATGTGA CGCTAAGTGT CAAACACCCC AAGGAGCTAT AAACAGTAGT CTCCCCTTCC 960

AGAATGTACA CCCAGTCACA ATAGGAGAGT GTCCAAAGTA TGTCAGGAGT ACAAAATTAA 1020

GGATGGTTAC AGGACTAAGG AACGTCCCAT CCATTCAATC CAGAGGTTTG TTTGGAGCCA 1080

TTGCCGGTTT CATTGAAGGA GGGTGGACTG GAATGATAGA TGGATGGTAT GGTTATCATC 1140

ATCAAAATGA ACAAGGATCT GGCTATGCTG CGGACCAAAA AAGCACACAA AATGCCATTA 1200

ATGGGATTAC AAACAAGGTG AATTCTATAA TCGAGAAAAT GAACACTCAA TTCACAGCTG 1260

TAGGCAAAGA ATTCAACAAA TTAGAAAAAA GGATGGAAAA CTTAAATAAG AAAGTTGATG 1320

ATGGATTTCT GGACATTTGG ACATATAATG CAGAATTGTT AGTTCTCCTG GAAAATGAAA 1380

GGACTTTGGA TTTTCATGAC TTAAATGTGA AGAACCTGTA TGAGAAAGTG AAAAACCAAT 1440

TGAAGAATAA TGCCAAAGAA ATAGGGAACG GGTGTTTTGA ATTCTATCAC AAGTGTAACA 1500

ATGAATGCAT GGAAAGTGTG AAAAATGGAA CTTATGACTA TCCAAAATAT TCCAAAGAAT 1560

CAAAGTTAAA CAGGGAAAAA ATTGATGGAG TGAAATTGGA ATCAATGGGA GTCTATCAGA 1620

TTCTGGCGAT CTACTCAACT GTCGCCAGTT CGCTGGTGCT TTTGGTCTCC CTGGGGGCAA 1680

TCAGCTTCTG GATGTGTTCT AATGGGTCTT TGCAGTGTAG AATATGCATC TGAGACCAGA 1740

ATTTCAGAAA TATAAGAAAA AACACCCTTG TTTCTACT 1778

**A_seg4_HA2**

AGCAAAAGCA GGGGTTATAC CATAGACAAC CAAAGGCAAG ACAATGGCCA TCATTTATCT 60

AATTCTTCTG TTCACAGCAG TGAGAGGGGA CCAAATATGC ATTGGATACC ATTCCAACAA 120

TTCCACAGAA AAGGTTGACA CAATCCTAGA GAGAAATGTC ACTGTGACTC ACGCTCAGGA 180

CATTCTTGAG AAGACTCACA ATGGGAAGTT ATGCAAACTA AATGGAATCC CTCCACTTGA 240

ATTAAGGGAT TGCAGCATTG CCGGATGGCT CCTTGGGAAT CCAGAATGTG ATATACTTCT 300

AACTGTGCCA GAATGGTCAT ACATAATAGA AAAAGAAAAT CCAAGGAACG GCTTGTGCTA 360

CCCAGGCAGT TTCAATGATT ATGAAGAATT GAAGCATCTT ATCAGCAGCG TGACACATTT 420

TGAGAAAGTA AAGATTCTGC CCAGAAATGA ATGGACACAG CATACAACAA CTGGAGGTTC 480

ACAGGCTTGC GCAGCATATG GTGGTCCGTC ATTCTTCCGG AACATGGTCT GGTTGACAAA 540

GAAAGGGTCG AATTATCCAA TTGCCAAAAG ATCTTACAAC AATACAAGTG GGGAACAAAT 600

GCTGATCATT TGGGGGATAC ATCACCCCAA TGATGAAAGT GAACAAAGAG CATTGTATCA 660

GAATGTGGGG ACCTATGTGT CAGTAGGAAC ATCAACACTG AACAAAAGAT CAATCCCAGA 720

AATAGCAACA AGACCTAAAG TGAATGGACA AGGAGGCAGA ATGGAATTCT CGTGGACTAT 780

CTTAGATATA TGGGACACAA TAAATTTTGA AAGTACAGGC AATCTAATTG CACCAGAATA 840

TGGTTTCAAA ATATCCAAAC GAGGTAGTTC AGGGATCATG AAAACAGAAG GAAAACTTGA 900

AAACTGCGAG ACCAAGTGCC AAACTCCTTT GGGAGCAATA AATACAACAT TACCCTTTCA 960

CAATATCCAC CCACTGACCA TTGGTGAGTG CCCCAAATAT GTAAAATCGG AAAGATTAGT 1020

CTTAGCAACA GGACTAAGAA ACGTCCCTCA GATTGAGTCA AGGGGATTGT TTGGGGCAAT 1080

AGCTGGTTTT ATAGAGGGTG GATGGCAAGG AATGGTTGAT GGTTGGTATG GGTATCATCA 1140

CAGCAATGAC CAGGGATCTG GGTATGCAGC AGACAAAGAA TCCACTCAAA AGGCAATTGA 1200

TGGAATCACC AACAAGGTAA ATTCTGTGAT CGAAAAGATA AACACCCAAT TCGAAGCTGT 1260

TGGAAAAGAA TTCAGTAACT TGGAGAGAAG ACTGGAGAAC TTGAATAAAA AGATGGAGGA 1320

CGGATTTCTA GATGTGTGGA CATACAATGC CGAGCTCCTA GTTCTAATGG AAAATGAGAG 1380

GACACTTGAC TTTCATGATT CTAATGTCAA GAATCTATAT GATAAAGTCA GAATGCAACT 1440

GAGAGACAAT GCAAAAGAAC TAGGGAATGG ATGTTTTGAA TTTTATCACA AATGTGATGA 1500

TGAATGCATG AACAGTGTGA AGAATGGGAC ATATGATTAT TCCAAGTATG AAGAGGAGTC 1560

TAAACTAAAC AGGACTGAAA TCAAAGGGGT TAAATTGAGC AATATGGGGG TTTATCAAAT 1620

CCTTGCCATC TATGCTACAG TAGCAGGTTC CCTGTCACTG GCAATCATGA TAGCTGGGAT 1680

TTCTATATGG ATGTGCTCCA ACGGGTCTCT GCAATGCAGA ATCTGCATAT GATTATCAGT 1740

CATTTTGTAA TTAAAAACAC CCTTGTTTCT ACT 1773

**A_seg4_HA3**

AGCAAAAGCA GGGGATAATT CTATTAACCA TGAAGACTAT CATTGCTTTG AGCTACATTC 60

TATGTCTGGT TTTCGCTCAA AAACTTCCCG GAAATGACAA CAGCACGGCA ACGCTGTGCC 120

TTGGGCACCA TGCAGTACCA AACGGAACGA TAGTGAAAAC AATCACGAAT GACCAAATTG 180

AAGTTACTAA TGCTACTGAG CTGGTTCAGA GTTCCTCAAC AGGTGGAATA TGCGACAGTC 240

CTCATCAGAT CCTTGATGGA GAAAACTGCA CACTAATAGA TGCTCTATTG GGAGACCCTC 300

AGTGTGATGG CTTCCAAAAT AAGAAATGGG ACCTTTTTGT TGAACGCAGC AAAGCCTACA 360

GCAACTGTTA CCCTTATGAT GTGCCGGATT ATGCCTCCCT TAGGTCACTA GTTGCCTCAT 420

CCGGCACACT GGAGTTTAAC AATGAAAGCT TCAATTGGAC TGGAGTCACT CAAAATGGAA 480

CAAGCTCTGC TTGCAAAAGG AGATCTAATA ACAGTTTCTT TAGTAGATTG AATTGGTTGA 540

CCCACTTAAA ATTCAAATAC CCAGCATTGA ACGTGACTAT GCCAAACAAT GAAAAATTTG 600

ACAAATTGTA CATTTGGGGG GTTCACCACC CGGGTACGGA CAATGACCAA ATCAGCCTAT 660

ATGCTCAAGC ATCAGGAAGA ATCACAGTCT CTACCAAAAG AAGCCAACAA ACTGTAATCC 720

CGAATATCGG ATCTAGACCC AGAGTAAGGG ATATCCCCAG CAGAATAAGC ATCTATTGGA 780

CAATAGTAAA ACCGGGAGAC ATACTTTTGA TTAACAGCAC AGGGAATCTA ATTGCTCCTC 840

GGGGTTACTT CAAAATACGA AGTGGGAAAA GCTCAATAAT GAGATCAGAT GCACCCATTG 900

GCAAATGCAA TTCTGAATGC ATCACTCCAA ATGGAAGCAT TCCCAATGAC AAACCATTTC 960

AAAATGTAAA CAGGATCACA TATGGGGCCT GTCCCAGATA TGTTAAGCAA AACACTCTGA 1020

AATTGGCAAC AGGGATGCGA AATGTACCAG AGAAACAAAC TAGAGGCATA TTTGGCGCAA 1080

TCGCGGGTTT CATAGAAAAT GGTTGGGAGG GAATGGTGGA TGGTTGGTAC GGTTTCAGGC 1140

ATCAAAATTC TGAGGGAATA GGACAAGCAG CAGATCTCAA AAGCACTCAA GCAGCAATCA 1200

ACCAAATCAA TGGGAAGCTG AATAGGTTGA TCGGGAAAAC CAACGAGAAA TTCCATCAGA 1260

TTGAAAAAGA ATTCTCAGAA GTAGAAGGGA GAATTCAGGA CCTCGAGAAA TATGTTGAGG 1320

ACACTAAAAT AGATCTCTGG TCATACAACG CGGAGCTTCT TGTTGCCCTG GAGAACCAAC 1380

ATACAATTGA TCTAACTGAC TCAGAAATGA ACAAACTGTT TGAAAGAACA AAGAAGCAAC 1440

TGAGGGAAAA TGCTGAGGAT ATGGGCAATG GTTGTTTCAA AATATACCAC AAATGTGACA 1500

ATGCCTGCAT AGGGTCAATC AGAAATGGAA CTTATGACCA TGATGTATAC AGAGATGAAG 1560

CATTAAACAA CCGGTTCCAG ATCAAAGGTG TTGAGCTGAA GTCAGGATAC AAAGATTGGA 1620

TCCTATGGAT TTCCTTTGCC ATATCATGTT TTTTGCTTTG TGTTGCTTTG TTGGGGTTCA 1680

TCATGTGGGC CTGCCAAAAA GGCAACATTA GGTGCAACAT TTGCATTTGA GTGCATTAAT 1740

TAAAAACACC CTTGTTTCTA CT 1762

**A_seg4_HA4**

AGCAAAAGCA GGGGAAACAA TGCTATCAAT CACGATTCTG TTTCTGCTCA TAGCAGAGGT 60

CTCCTCTCAG AACTACACAG GAAACCCTGT GATATGCCTA GGACACCATG CTGTATCCAA 120

TGGGACAATG GTGAAAACCT TGACTGATGA CCAAGTAGAA GTCGTCACTG CCCAGGAATT 180

AGTGGAATCG CAACATCTAC CGGAGTTGTG TCCTAGCCCT TTAAGATTAG TAGATGGGCA 240

AACTTGTGAC ATCGTCAATG GTGCTCTGGG GAGCCCAGGC TGTGATCACT TGAATGGTGC 300

AGAATGGGAT GTCTTCATAG AGCGACCCAC TGCTGTGGAC ACTTGTTATC CATTTGATGT 360

GCCAGATTAC CAGAGCCTAC GGAGTATCTT AGCAAACAAT GGGAAATTTG AGTTCATTGC 420

AGAGGAATTC CAATGGAACA CAGTCAAGCA GAATGGGAAA TCCGGAGCAT GCAAAAGAGC 480

AAATGTGAAT GACTTTTTCA ACAGGCTGAA CTGGCTGACC AAATCTGATG GGAATGCATA 540

CCCACTCCAA AACCTGACAA AGGTCAACAA CGGGGACTAT GCAAGGCTTT ACATATGGGG 600

AGTTCACCAT CCTTCAACTG ACACAGAACA AACCAACTTA TATAAGAACA ACCCTGGAAG 660

AGTGACCGTC TCTACCAAAA CCAGTCAAAC AAGTGTGGTA CCAAATATTG GCAGTAGACC 720

ATGGGTGAGA GGCCAAAGCG GCAGGATCAG CTTCTATTGG ACAATTGTAG AACCAGGAGA 780

TCTCATAGTT TTCAACACCA TAGGGAATTT AATCGCTCCG AGGGGCCATT ACAAACTCAA 840

CAGCCAAAAG AAGAGCACAA TTCTGAATAC TGCAGTTCCT ATAGGATCCT GCGTTAGTAA 900

ATGCCACACC GACAGGGGTT CAATCACTAC AACTAAACCC TTTCAAAACA TCTCGAGAAT 960

ATCAATCGGG GACTGTCCCA AGTATATCAA ACAGGGATCT TTGAAACTAG CTACAGGGAT 1020

GAGGAACATC CCTGAGAAGG CAACCAGAGG TCTATTTGGT GCAATTGCTG GCTTTATAGA 1080

GAATGGCTGG CAAGGTCTAA TTGATGGTTG GTATGGGTTT AGGCACCAGA ATGCAGAAGG 1140

GACGGGGACA GCTGCAGATC TCAAGTCGAC TCAGGCAGCT ATTGATCAAA TTAATGGAAA 1200

ATTGAATCGT CTCATTGAGA AAACAAATGA GAAATACCAC CAAATTGAAA AGGAATTTGA 1260

ACAAGTAGAG GGAAGAATCC AAGACTTAGA AAAGTATGTT GAAGACACAA AGATTGACCT 1320

GTGGTCTTAC AATGCTGAAT TATTGGTGGC ATTAGAAAAT CAACATACTA TAGATGTGAC 1380

AGACTCCGAA ATGAACAAAC TCTTTGAAAG AGTTAGGCGC CAACTAAGAG AGAATGCTGA 1440

AGACAAAGGA AATGGATGTT TTGAAATTTT CCATCAGTGT GACAACAACT GCATTGAAAG 1500

CATAAGGAAT GGAACATATG ACCATGATAT TTACAGAGAC GAGGCAATCA ATAACAGATT 1560

CCAGATACAA GGAGTTAAAT TGACTCAAGG ATACAAGGAC ATTATTCTCT GGATTTCCTT 1620

TTCCATATCA TGCTTCTTAC TCGTTGCACT ACTTTTAGCC TTTATTTTGT GGGCTTGTCA 1680

GAATGGAAAC ATCCGGTGCC AGATTTGCAT TTAAAGAAAA AACACCCTTG TTTCTACT 1738

**A_seg4_HA5**

AGCAAAAGCA GGGGTTCAAT CTGTCAAAAT GGAGAAAATA GTGCTTCTTC TTGCAATAGT 60

CAGTCTTGTT AAAAGTGATC AGATTTGCAT TGGTTACCAT GCAAACAACT CGACAGAGCA 120

GGTTGACACA ATAATGGAAA AGAACGTCAC TGTTACACAC GCCCAAGACA TACTGGAAAA 180

GACACACAAC GGGAAGCTCT GCGATCTAGA TGGAGTGAAG CCCCTAATTT TAAGAGATTG 240

TAGTGTAGCT GGATGGCTCC TCGGGAACCC AATGTGTGAC GAATTCCTCA ATGTGCCGGA 300

ATGGTCTTAC ATAGTGGAGA AGATCAATCC AGCCAATGAC CTCTGTTACC CAGGGAATTT 360

CAACGACTAT GAAGAACTGA AACACCTATT GAGCAGAATA AACCATTTTG AGAAAATTCA 420

GATCATCCCC AAAAGTTCTT GGTCAGATCA TGAAGCCTCA TCAGGGGTGA GCTCAGCATG 480

TCCATACCAG GGAAGGTCCT CCTTTTTTAG AAATGTGGTA TGGCTTATCA AAAAGAACAA 540

TGCATACCCA ACAATAAAGA GAAGTTACAA TAATACCAAC CAAGAAGATC TTTTGGTACT 600

GTGGGGGATT CACCATCCAA ATGATGCGGC AGAGCAGACA AGGCTCTATC AAAACCCAAC 660

CACCTATATT TCCGTTGGGA CATCAACACT AAACCAGAGA TTGGTACCGA AAATAGCTAC 720

TAGATCCAAA GTAAACGGGC AAAGTGGAAG GATGGAGTTC TTTTGGACAA TTTTAAAACC 780

GAATGATGCA ATAAACTTTG AGAGTAATGG AAATTTCATT GCTCCAGAAA ATGCATACAA 840

AATTGTCAAG AAAGGGGACT CAACAATTAT GAAAAGTGAA TTGGAATATG GTAACTGCAA 900

CACCAAGTGT CAAACTCCAA TAGGGGCGAT AAACTCTAGT ATGCCATTCC ACAACATCCA 960

CCCTCTCACC ATCGGGGAAT GCCCCAAATA TGTGAAATCA AACAGATTAG TCCTTGCGAC 1020

TGGGCTCAGA AATAGCCCTC AAGGAGAGAG AAGAAGAAAA AAGAGAGGAC TATTTGGAGC 1080

TATAGCAGGT TTTATAGAGG GAGGATGGCA GGGAATGGTA GATGGTTGGT ATGGGTACCA 1140

CCATAGCAAC GAGCAGGGGA GTGGGTACGC TGCAGACAAA GAATCCACTC AAAAGGCAAT 1200

AGATGGAGTC ACCAATAAGG TCAACTCGAT CATTGACAAA ATGAACACTC AGTTTGAGGC 1260

TGTTGGAAGG GAATTTAATA ACTTAGAAAG GAGAATAGAA AATTTAAACA AGAAGATGGA 1320

AGACGGATTC CTAGATGTCT GGACTTATAA TGCTGAACTT CTGGTTCTCA TGGAAAATGA 1380

GAGAACTCTA GACTTTCATG ACTCAAATGT CAAGAACCTT TACGACAAGG TCCGACTACA 1440

GCTTAGGGAT AATGCAAAGG AGCTTGGTAA CGGTTGTTTC GAGTTCTATC ACAGATGTGA 1500

TAATGAATGT ATGGAAAGTG TAAGAAACGG AACGTATGAC TACCCGCAGT ATTCAGAAGA 1560

AGCAAGATTA AAAAGAGAGG AAATAAGTGG AGTAAAATTG GAATCAATAG GAACTTACCA 1620

AATACTGTCA ATTTATTCAA CAGTAGCGAG CTCCCTAGCA CTGGCAATCA TGGTGGCTGG 1680

TCTATCTTTA TGGATGTGCT CCAATGGATC GTTACAATGC AGAATTTGCA TTTAAATTTG 1740

TGAGTTCAGA TTGTAGTTAA AAACACCCTT GNTTCCTACT 1780

**A_seg4_HA6**

AGCAAAAGCA GGGGAAAATG ATTGCAATCA TTGTAATAGC GATACTGGCA TCAGCCGGAA 60

AGTCAGACAA GATCTGCATT GGGTATCACG CCAACAATTC AACAACACAA GTGGATACGC 120

TACTTGAGAA GAATGTAACC GTCACACACT CAGTTGAATT GCTGGAGAGT CAGAAGGAAA 180

AGAGATTCTG CAAGATTATG AACAAGGCCC CTCTCGATCT AAGGGATTGC ACCATAGAGG 240

GTTGGATCTT GGGGAATCCT AAATGCGACT TATTGCTTGG TGATCAAAGC TGGTCATACA 300

TAGTGGAAAG GCCTACTGCT CAAAATGGGA TTTGCTACCC AGGAGTTTTG AATGAAGTAG 360

AAGAACTGAA GGCACTTATA GGATCAGGAG AAAGAGTAGA GAGATTTGAG ATGTTTCCCA 420

AAAGTACATG GGCAGGGGTA GACACCAGCA GGGGGGTGAC AAACGCTTGC CCTTCCTATA 480

CTATTGATTC ATCTTTCTAC AGAAACCTCG TATGGATAGT AAAAACTGAG TCAGCAGCAT 540

ATCCAGTAAT TAAAGGGACT TACAACAACA CTGGAAACCA GCCAATCCTT TATTTCTGGG 600

GTGTTCACCA TCCTCCTGAC ACCACGGTGC AAGATAACTT GTATGGGTCT GGTGATAGAT 660

ACGTTAGAAT GGGAACTGAA AGCATGAACT TTGCCAAGAG TCCGGAAATT GCAGCAAGAC 720

CTGCTGTGAA CGGTCAAAGG AGCAGAATTG ATTATTACTG GTCTGTTTTG AAACCAGGGG 780

AAACCTTGAA TGTGGAGTCC AATGGAAATC TAATCGCTCC TTGGTATGCA TACAAATTTG 840

TCAGCACAAA TAAAAAGGGA GCCGTCTTCA AGTCAAATTT ACCAATCGAG AACTGCGATG 900

CCACATGCCA GACTATTGCA GGGGTCCTAA GGACCAATAA AACATTTCAG AATGTGAGCC 960

CTCTGTGGAT AGGAGAATGC CCCAAATATG TGAAAAGTGA AAGTCTGAGG CTTGCAACTG 1020

GACTGAGAAA TGTTCCACAG ATTGAAACTA GAGGAATTTT CGGAGCTATC GCAGGTTTTA 1080

TTGAAGGAGG ATGGACTGGA ATGATAGATG GGTGGTATGG CTATCACCAT GAAAATTCTC 1140

AAGGCTCAGG ATATGCAGCA GACAGAGAAA GCACTCAAAA GGCTATAGAC GGAATTACAA 1200

ATAAGGTCAA TTCCATCATC AACAAAATGA ACACACAATT CGAAGCTGTA GACCACGAAT 1260

TTTCAAATCT GGAGAGAAGA ATTGGCAATC TGAACAAGAG AATGGAAGAT GGATTTCTGG 1320

ATGTTTGGAC ATACAATGCT GAACTGTTGG TTCTTCTTGA AAATGAAAGA ACACTAGATC 1380

TGCATGATGC GAATGTAAAG AACCTATATG AAAAGGTCAA ATCACAGCTA AGAGACAATG 1440

CTAATGACCT AGGAAATGGG TGCTTTGAAT TTTGGCATAA ATGTGACAAT GAATGCATAG 1500

AGTCTGTCAA AAATGGTACC TATGACTATC CCAAATACCA GGATGAGAGT AAATTAAACA 1560

GGCAGGGAAT AGAATCGGTG AAGCTAGAGA ATCTTGGTGT GTATCAAATT CTTGCTATTT 1620

ATAGTACGGT ATCGAGCAGT CTAGTTTTGG TAGGACTGAT CATGGCAATA GGTCTTTGGA 1680

TGTGTTCAAA TGGTTCAATG CAGTGCAGAA TATGTATATA GTTAAGAAAA ACACCCTTGT 1740

TTCTACT 1747

**A_seg4_HA7**

AGCAAAAGCA GGGGATACGA AATGAACACT CAAATCCTGG TATTCGCTCT GGTGGCGATC 60

ATTCCGACAA ATGCAGACAA AATCTGCCTT GGGCATCATG CCGTGTCAAA CGGGACTAAA 120

GTAAACACAT TAACTGAAAG AGGAGTGGAA GTCGTTAATG CAACTGAAAC GGTGGAACGA 180

ACAAACGTCC CCAGGATCTG CTCAAAAGGG AAAAGGACAG TTGACCTCGG TCAATGTGGA 240

CTTCTGGGAA CAATCACTGG GCCACCCCAA TGTGACCAAT TCCTAGAATT TTCAGCCGAT 300

CTAATTATTG AGAGGCGAGA AGGAAGTGAT GTCTGTTACC CTGGGAAATT CGTGAATGAA 360

GAAGCTCTGA GGCAAATTCT CAGGGAGTCA GGCGGAATTG ACAAGGAGAC AATGGGATTC 420

ACATACAGCG GAATAAGAAC TAATGGAGCA ACCAGTTCAT GTAGGAGATC AGGATCTTCA 480

TTCTATGCAG AGATGAAATG GCTCCTGTCA AACACAGACA ATGCTGCTTT CCCGCAGATG 540

ACTAAGTCAT ACAAAAACAC AAGGAAAGAC CCAGCTCTGA TAATATGGGG GATCCACCAT 600

TCCGGATCAA CTACAGAACA GACCAAGCTA TATGGGAGTG GAAACAAACT GATAACAGTT 660

GGGAGTTCTA ATTACCAACA GTCCTTTGTA CCGAGTCCAG GAGCGAGACC ACTAGTGAAT 720

GGCCAATCTG GAAGAATTGA CTTTCATTGG CTGATGCTAA ACCCCAATGA CACAGTCACT 780

TTCAGTTTCA ATGGGGCCTT CATAGCTCCA GACCGTGCAA GTTTTCTGAG AGGGAAGTCT 840

ATGGGGATTC AGAGTGGAGT ACAGGTTGAT GCCAATTGTG AAGGAGATTG CTATCACAGT 900

GGAGGGACAA TAATAAGTAA TTTGCCCTTT CAGAACATAA ATAGCAGGGC AGTAGGGAAA 960

TGTCCGAGAT ATGTTAAGCA AGAGAGTCTG CTGCTGGCAA CAGGGATGAA GAATGTTCCC 1020

GAAGTTCCAA AGGGAAGAGG CCTATTTGGT GCTATAGCGG GTTTCATTGA AAATGGATGG 1080

GAAGGTCTGA TTGATGGGTG GTATGGCTTC AGGCATCAAA ATGCACAAGG GGAGGGAACT 1140

GCTGCAGATT ACAAAAGCAC CCAATCAGCA ATTGATCAAG TAACAGGAAA ATTGAACCGG 1200

CTTATAGAAA AAACTAACCA ACAATTTGAG TTAATAGACC ATGAATTCAC TGAGGTTGAA 1260

AAGCAAATTG GCAATGTGAT AAATTGGACC AGAGATTCCA TGACAGAAGT GTGGTCCTAT 1320

AACGCTGAAC TCTTGGTAGC AATGGAAAAC CAGCATACAA TTGATCTGGC CGACTCAGAA 1380

ATGAACAAAC TATACGAACG AGTGAAGAGA CAACTGAGAG AGAATGCTGA AGAAGATGGC 1440

ACTGGTTGCT TCGAAATATT TCACAAGTGT GATGACGACT GTATGGCCAG TATTAGAAAC 1500

AACACCTATG ATCACAGCAA GTACAGGGAA GAGGCAATGC AAAATAGAAT ACAGATTGAC 1560

CCAGTCAAAC TAAGCAGCGG CTACAAAGAT GTGATACTTT GGTTTAGCTT CGGGGCATCA 1620

TGTTTCATAC TTCTGGCCAT TGCAATGGGC CTTGTCTTCA TATGTGTGAA GAATGGAAAC 1680

ATGCGGTGCA CTATTTGTAT ATAAGTTTGG AAAAAACACC CTTGTTTCTA CT 1732

**A_seg4_HA8**

AGCAAAAGCA GGGGTCACAA TGGAGAAATT CATCGCAATA GCAATGCTCT TGGCGAGCAC 60

AAATGCATAC GATAGAATAT GCATTGGATA CCAATCAAAC AATTCCACAG ACACAGTGAA 120

CACTCTCATA GAACAAAATG TGCCAGTCAC CCAAACAATG GAGCTCGTAG AAACAAAGAA 180

ACATCCCGCT TATTGTAACA CTGATTTAGG TGCTCCACTG GAACTGCGAG ACTGCAAGAT 240

CGAGGCAGTA ATCTATGGGA ACCCCAAATG TGACATTCAT CTGAAAGATC AAGGTTGGTC 300

ATACATAGTG GAGAGGCCTA GCGCACCAGA AGGGATGTGT TACCCTGGAT CTGTGGAAAA 360

TCTGGAAGAA CTGAGGTTTG TCTTCTCCAG TGCAGCATCT TACAAGAGAA TAAGGCTATT 420

TGATTATTCC AGGTGGAATG TGACTCGTTC TGGAACAAGT AAAGCATGCA ATGCATCAAC 480

AGGTGCCCAA TCCTTCTATA GAAGCATCAA CTGGTTGACC AAAAAGAAAC CAGACACTTA 540

TGACTTCAAT GAAGGGACCT ATGTTAACAA TGAAGATGGA GACATCATTT TCTTATGGGG 600

GATCCACCAT CCTCCAAACA CAAAAGAGCA AACAACACTA TATAAGAATG CAAACACTTT 660

GAGTAGTGTT ACTACCAACA CTATAAACAG AAGCTTTCAA CCAAATATTG GTCCCAGACC 720

ATTAGTAAGA GGACAGCAAG GGAGAATGGA TTACTATTGG GGCATTCTGA AGAGAGGAGA 780

AACTCTGAAG ATCAGGACCA ATGGAAATTT AATCGCACCT GAATTTGGCT ATTTGCTCAA 840

AGGTGAAAGC CATGGCAGAA TAATCCAAAA CGAGGATATA CCCATTGGGA ATTGTAACAC 900

AAAATGTCAA ACATATGCGG GAGCAATCAA TAGCAGCAAA CCCTTTCAGA ATGCAAGTAG 960

GCATTACATG GGAGAATGTC CCAAATATGT GAAGAAGGCT AGCCTGCGAC TTGCAGTTGG 1020

GCTTAGGAAT ACGCCTTCTG TTGAACCCAA AGGACTGTTC GGAGCCATTG CTGGTTTCAT 1080

TGAAGGAGGA TGGTCTGGGA TGATTGATGG GTGGTATGGA TTTCATCACA GCAATTCAGA 1140

GGGAACAGGA ATGGCAGCTG ACCAGAAATC AACACAAGAA GCCATCGATA AGATAACCAA 1200

TAAAGTCAAC AATATAGTTG ACAAAATGAA CAGGGAATTT GGAGTTGTGA ATCATGAGTT 1260

CTCAGAAGTT GAAAAAAGGA TAAACATGAT AAACGATAAA ATAGATGACC AAATTGAAGA 1320

CCTTTGGGCT TACAATGCAG AGCTCCTTGT GCTCTTAGAG AACCAGAAAA CGCTAGACGA 1380

ACATGATTCC AATGTCAAAA ACCTTTTCGA TGAAGTGAAG AGGAGACTGT CAGCCAATGC 1440

AATAGATGCT GGGAACGGTT GCTTTGACAT ACTTCACAAA TGCGACAATG AGTGTATGGA 1500

AACTATAAAG AACGGAACTT ACGATCACAA GGAATATGAA GAGGAAGCTA AACTAGAAAG 1560

GAGCAAGATA AATGGAGTAA AACTAGAAGA GAATACCACT TACAGAATTC TTAGCATTTA 1620

CAGTACAGTG GCGGCCAGTC TTTGCTTGGC AATCCTGATT GCTGGAGGTT TAATCCTGGG 1680

CATGCAAAAT GGATCTTGTA GATGCATGTT CTGTATTTGA AGAAAAAACA CCCTTGTTTC 1740

TACT 1744

**A_seg4_HA9**

AGCAAAAGCA GGGGAATTTC ACAATTAAAC AAAATGGAAA TAACAATACT AATAGCTATA 60

CTGCTAATGG TAACAGCAAG CAATGCGGAT AAAATCTGCA TAGGCTACCA GTCAACAAAT 120

TCCACAGAAA CTGTTGACAC GCTAATAGAA AACAATGTTC CTGTGACACA TACCAAAGAA 180

TTGCTCCACA CAGAGCACAA TGGAATGCTG TGTGCAACAA ATCTGGGGCA TCCTCTCATT 240

CTAGACACCT GTACTATCGA AGGGCTGGTC TATGGCAATC CTTCTTGTGA TCTGCTATTG 300

GGAGGGAAAG AATGGTCCTA CATTGTCGAG AGATCATCTG CTGTCAATGG AATGTGTTAC 360

CCTGGGAATG TAGAAAACCT GGAAGAACTC AGGTCCTTTT TCAGTTCCGC TAATTCCTAC 420

CAAAGAATCC AGATCTTTCC AGACACAATC TGGAATGTGA CTTACAGTGG AACAAGCAAA 480

GCATGTTCAA ATTCATTCTA CAGGAGTATG AGATGGCTGA CTCACAAGAG CAATTCTTAT 540

CCCTTTCAAG ACGCCCAATA CACCAACAAT GAGGGGAAGA ACATTCTCTT CATGTGGGGC 600

ATACACCATC CACCTACTGA TGCCGAACAG ACAAACTTAT ACAAAAAGGC TGATACAACA 660

ACAAGTGTGA CAACAGAAGA TATAAATCGG ACTTTCAAGC CAGTGATAGG GCCAAGGCCC 720

CTTGTCAATG GACAGCAGGG GAGAATTGAC TATTATTGGT CAGTATTAAA ACCAGGCCAA 780

ACGTTGCGAG TGAGATCCAA TGGAAACCTA ATTGCCCCAT GGTATGGTCA CATTCTCTCA 840

GGAGAAAGCC ATGGAAGAAT CTTGAAGACT GATTTGAGCA GTGGCGACTG CGTAGTGCAG 900

TGTCAGACTG AGAAAGGTGG TCTAAATACG ACCTTGCCGT TTCATAATGT CAGCAAATAT 960

GCATTTGGGA ATTGCCCCAG ATATGTTGGA GTGAAGAGTC TCAAACTGGC AGTCGGTTTA 1020

AGGAATGTAC CTGCTGTATC AGATAGAGGG CTGTTTGGTG CTATAGCTGG ATTCATAGAA 1080

GGAGGTTGGC CAGGGCTAGT TGCTGGCTGG TATGGGTTCC AGCATTCAAA TGACCAAGGA 1140

ACTGGAATGG CTGCAGATAG AGAGTCAACT CAAGAAGCAG TTGACAAAAT AACATCCAAA 1200

GTGAATAATA TAATAGACAA AATGAACAAG CAATATGAGA TCATTGATCA TGAATTCAGT 1260

GAAATTGAAG CCAGGCTCAA CATGATCAAC AACAAGATTG ATGACCAAAT ACAAGACATT 1320

TGGGCATACA ATGCAGAACT GCTAGTGCTG CTTGAAAATC AGAAAACACT CGATGAACAT 1380

GATGCGAATG TAAATAATCT ATATAACAAG GTGAAAAGAG CACTGGGTTC TAATGCAGCA 1440

GAAGATGGGA ATGGATGCTT CGAATTGTAC CATAAATGTG ATGATCAATG CATGGAAACG 1500

ATTAGAAACG GGACCTATGA CAGGCAAAAG TATCAAGAGG AGTCAAAATT AGAGAGACAG 1560

AAAATAGAAG GGGTAAAACT GGAATCTGAA GGAACTTACA AAATCCTCAC TATTTATTCG 1620

ACTGTCGCCT CATCTCTTGT GCTTGCAATG GGGTTTGCTG CCTTTCTGTT CTGGGCCATG 1680

TCCAATGGAT CTTGCAGATG CAACATTTGT ATATAATTAG CAAAAACACC CTTGTTTCTA 1740

CT 1742

**A_seg4_H10**

AGCAAAAGCA GGGGTCACAA TGTACAAAAT AGTACTAGTA CTTGCGCTCC TTGGAGCGGT 60

GCATGGTCTT GACAAAATAT GCCTTGGACA TCATGCAGTC CCCAATGGCA CCATCGTAAA 120

GACTCTCACA AACGAAAAGG AAGAGGTGAC CAATGCTACT GAAACGGTGG AAAGCAAAAG 180

CCTGGACAAA CTTTGCATGA AAAGTCGGAA TTACAAGGAC CTAGGTAATT GCCACCCGAT 240

AGGGATGGTA ATAGGGACTC CTGCTTGTGA CCTACACCTC ACCGGAACAT GGGACACTTT 300

AATAGAGAGA GACAATTCTA TTGCCTACTG TTACCCGGGT GCCACTGTGA ATGAAGAAGC 360

ATTAAGGCAG AAAATTATGG AAAGTGGAGG GATTGACAAG ATAAGCACCG GGTTTACATA 420

TGGATCATCC ATCAATTCAG CTGGAACCAC TAGATCATGC ATGAGAAGTG GAGGAAACAG 480

TTTCTATGCG GAGCTAAAGT GGCTAGTGTC GAAGAACAAA GGACAAAACT TCCCACAAAC 540

AGCAAACACA TACAGGAATA CAGATTCAGC AGAACACCTT ATAATCTGGG GAATTCATCA 600

CCCTTCAAGC ACACAAGAAA AGAATGATCT GTATGGAACA CAATCACTTT CCATTTCAGT 660

AGGGAGTTCT ACTTATCAAA ACAACTTTGT GCCTGTGGTG GGAGCAAGAC CACAAGTGAA 720

TGGCCAAAGT GGGCGTATTG ATTTCCATTG GACGATGGTA CAACCAGGTG ATAACATCAC 780

TTTTTCGCAT AACGGCGGAT TAATAGCACC TAGTAGAGTG AGTAAACTAA AGGGAAGAGG 840

CCTTGGCATT CAATCAGGAG CTTCAGTAGA TAATGACTGT GAATCAAAAT GTTTTTGGAA 900

AGGTGGATCC ATCAACACCA AACTCCCTTT TCAGAATCTT TCCCCAAGAA CTGTGGGTCA 960

ATGTCCCAAG TATGTGAACA AAAAGAGCCT GTTGCTTGCT ACCGGAATGA GGAATGTGCC 1020

AGAGGTTGTC CAAGGAAGAG GCCTGTTTGG AGCAATAGCT GGATTCATAG AAAATGGATG 1080

GGAAGGGATG GTAGATGGTT GGTATGGTTT CCGACATCAA AATGCCCAAG GCACTGGTCA 1140

GGCTGCGGAT TACAAAAGTA CTCAGGCAGC TATAGATCAA ATAACCGGGA AATTGAACAG 1200

GCTGATAGAG AAGACAAACA CAGAGTTCGA ATCCATAGAA TCTGAGTTCA GTGAAATAGA 1260

ACATCAAATT GGCAATGTAA TAAACTGGAC TAAGGATTCT ATAACAGACA TTTGGACGTA 1320

TCAAGCTGAA TTGCTGGTAG CAATGGAAAA TCAGCACACA ATTGACATGG CTGATTCAGA 1380

AATGCTGAAT CTATACGAAA GGGTGAGGAA GCAACTAAGG CAAAATGCAG AAGAAGATGG 1440

AAAAGGGTGC TTTGAAATAT ATCACAAATG CGACGACAAC TGCATGGAAA GCATCAGAAA 1500

CAACACCTAT GACCATACAC AATACAGAGA AGAAGCACTC TTGAACAGAC TCAACATTAA 1560

TCCGGTGAAA CTCTCTTCTG GGTACAAAGA TGTTATACTG TGGTTTAGCT TCGGGGCGTC 1620

ATGCTTTGTA CTTTTGGCTG TCATCATGGG GCTTGTTTTC TTCTGTTTGA AAAATGGAAA 1680

CATGCGATGC ACAATCTGTA TTTAGTTAAA AACACCCTTG TTTCTACT 1728

**A_seg4_H11**

AGCAAAAGCA GGGGAAATAT CAAGAAATCA AGATGAAGAA AGCAATCCTC CTTGCAGCAA 60

TCATCATCTG TATCCAAGCA GATGAGATCT GTATTGGGTA CCTGAGCAAC AATTCAACAG 120

AAAAAGTGGA CACAATAATT GAGAGCAACG TCACCGTTAC TAGCTCCGTT GAACTGGTTG 180

AAAATGAGCA CACCGGATCT TTTTGCTCGA TTGACGGGAA AGCACCGATA AGTCTTGGTG 240

ATTGCTCCTT TGCTGGATGG ATCCTTGGAA ACCCAATGTG TGATGATTTG ATTGGGAAAA 300

CGTCATGGTC CTACATAGTG GAGAAACCAA ATCCCGCTAA CGGAATCTGT TACCCGGGTA 360

GTCTAGACAA CGAAGAGGAA TTGAGACTGA AGTTTAGTGG GGTCCTCGAA TACAGCAAAT 420

TTGAAGCATT CACTTCAAAT GGATGGGGGG CGGTAAACTA TGGTGCTGGT GTTACCGCAG 480

CCTGCAAATA TGGAAGCAGT AACTCTTTTT TCAGGCACAT GGTATGGTTG ATCCACCAAT 540

CAGGGACATA TCCTGTTATA CGGAGGACAT TCAACAACAC CAAAGGAAGA GATGTATTAA 600

TGGTATGGGG AGTTCACCAT CCTTCAAGTC TACAGGAACA CCGAGACTTG TACAAAAAAG 660

ACATTTCTTA TGTAGCAGTG GGTTCAGAGA GCTACAACAG GAGGTTCACT CCTGAGATCA 720

GTGCGAGGCC TGCAGTAAAT GGTCAGGCTG GGAGAATGAC ATTCTATTGG ACCATAGTGA 780

AACCAAGGGA AACAATAACA TTTGAGTCAA ATGGTGCATT TATCGCTCCT CGGTATGCTT 840

TTGAGTTGGT GTCCTTGGGG AATGGGAAAC TGTTCAGGAG TGACCTGAAT ATTGAATCTT 900

GCTCAACTAG ATGCCAGTCA GAGATTGGAG GGATCAACAC TAACAAGAGC TTCCACAATG 960

TTCATAGAAA CACAATAGGA GACTGCCCCA AGTATGTGAA TGTTAAATCT CTAAAGCTTG 1020

CCACCGGACT CAGAAATATT CCTGCAATTG CCACAAGAGG CCTGTTTGGT GCAATAGCTG 1080

GTTTCATAGA AGGTGGTTGG CCAGGGTTAA TCAATGGTTG GTATGGGTTC CAACATAGAA 1140

ATGAAGAAGG TACAGGGATT GCTGCAGACA GAGAATCAAC CCAGAAGGCA ATAGACCAGA 1200

TAACATCTAA AGTCAATAAC ATTGTTGATC GGATGAATAC AAACTTTGAG TCTGTTCAAC 1260

ATGAATTCAG TGAAATTGAA GAGAGAATAA ACCAATTGTC GGCACGTGTG GATGATTCTG 1320

TTATTGATAT ATGGTCATAC AACGCACAGC TTCTTGTCCT ATTGGAGAAT GAAAAAACAT 1380

TAGATCTCCA TGATTCTAAT GTCAGGAACC TCCATGAAAA GGTCAGACGG ATGCTGAAAG 1440

ATAATGCTAA AGATGAAGGA AATGGTTGTT TTACTTTCTA CCACAAGTGT GATAATGAAT 1500

GCATTGAAAA GGTCAGGAAT GGGACATATG ACCACAAGGA ATTTGAAGAG GAGTCCAAGT 1560

TGAACAGACA AGAAATTGGA GGAGTAAAGC TGGATTCCAG TGGCAACGTT TACAAAATAC 1620

TGTCGATTTA CAGCTGCATT GCAAGTAGTC TTGTGTTGGC AGCAATCATT ATGGGGTTCA 1680

TTTTCTGGGC GTGTAGCAAT GGGTCATGTA GATGTACCAT TTGCATTTAG ACTTTCAGTA 1740

AAAACACCCT TGTTTCTACT 1760

**A_seg4_H12**

AGCAAAAGCA GGGGTCACAA TGGAAAAATT CATCATTTTG AGCACTGTCC TGGCAGTAAG 60

CCTTGCATAT GACAAAATTT GCATTGGATA TCAAACAAAC AACTCGACTG AAACGGTGAA 120

CACACTAATT GAACAAAATG TTCCGGTGAC ACAAGTGGAA GAACTCGTGC ATGGCGGAAT 180

TGACCCGATC CTATGTGGAA CGGAATTAGG ATCACCACTA GTGCTCGATG ACTGTTCATT 240

AGAGGGTCTA ATTCTGGGCA ATCCCAAATG TGATCTATAT CTGAATGGCA GAGAATGGTC 300

ATACATAGTA GAGAGGCCAA AAGAGATTGA AGGAATTTGC TATCCAGGAT CAATTGAAAA 360

CCAGGAAGAG CTAAGATTTC TGTTTTCTTC CATCAAAAAG TATGAAAGAG TGAAGATGTT 420

TGATTTCACC AAATGGAATG TCACATACAC TGGGACCAGC AAGGCATGCA ATGATACATC 480

AAACCAAGGC TCATTCTATA GGAGCATGAG ATGGTTGACA TTAAAGTCAG GACAATTCCC 540

AGTCCAGACA GATGAGTACA AGAACACCAG AGATTCAGAC ATTGTATTTA CATGGGCCAT 600

TCACCACCCA CCAACATCTG ATGAACAAGT AAAATTATAC AAAAATCCTG ATACCCTCTC 660

CTCAGTCACC ACTGATGAGA TCAATAGGAG CTTCAAGCCT AATATTGGGC CAAGGCCACT 720

CGTGAGAGGA CAACAGGGGA GAATGGATTA CTACTGGGCT GTCCTCAAAC CTGGACAAAC 780

TGTCAAAATA CAAACCAATG GTAATCTTAT TGCCCCTGAA TATGGTCATT TAATCACAGG 840

GAAATCACAT GGCAGGATAC TCAAGAATAA TTTGCCCATA GGGCAGTGTG TGACTGAGTG 900

CCAATTGAAT GAGGGTGTAA TGAATACGAG CAAACCTTTC CAGAACACTA GTAAGCACTA 960

TATTGGGAAA TGCCCCAAAT ACATACCATC AGGGAGTCTA AAACTGGCAA TAGGACTCAG 1020

AAATGTCCCA CAAGCTCAAG ATCGAGGACT CTTTGGAGCA ATTGCAGGCT TCATAGAAGG 1080

CGGATGGCCA GGACTAGTGG CTGGCTGGTA TGGATTTCAG CATCAAAATG CAGAGGGGAC 1140

AGGCATAGCT GCAGACAGAG ACAGCACTCA AATGGCAATA GACAATATGC AAAACAAACT 1200

CAACAATGTC ATCGACAAAA TGAACAAACA ATTTGAGGTG GTGAACCATG AGTTTTCAGA 1260

AGTGGAGAGC AGAATAAACA TGATTAATTC CAAAATTGAT GATCAGATAA CCGATATATG 1320

GGCATACAAT GCAGAATTGC TTGTTCTATT GGAAAATCAG AAGACATTAG ATGAGCATGA 1380

CGCTAATGTA AGGAATCTAC ATGATAGAAT CAGAAGAGTC CTGAGGGAAA ATGCAATTGA 1440

CACAGGAGAT GGATGTTTTG AAATTCTACA TAAATGTGAC AACAATTGCA TGGACACAAT 1500

CAGAAACGGA ACATACAATC ACAAAGAATA TGAGGAAGAA AGCAAAATTG AACGACAGAA 1560

AATCAATGGT GTGAAACTTG AGGAGAACTC TACATATAAA ATTCTGAGCA TCTACAGCAG 1620

TGTTGCCTCA AGCTTAGTTC TACTGCTCAT GATTATTGGG GGTTTCATTT TCGGGTGTCA 1680

AAATGGAAAT GTTCGTTGTA CTTTCTGTAT TTAATTAAAA ACACCCTTGT TTCTACT 1737

**A_seg4_H13**

AGCAAAAGCA GGGGAAATAT TTATAAATCG AAACAAGAAA ATGGATATCA GATCAATCGT 60

AATCTCGTTA CTTATTAGCA CGTGCGTACA GGCCGACAGA ATCTGTGTAG GATACTTAAG 120

CACCAACTCA ACAGAAAAAG TTGATACACT GTTGGAGAAT GATGTTCCAG TCACAAGCTC 180

TATTGATCTA GTTGAGACAA ACCACACAGG AACATATTGT TCTTTGGATG GAGTCAGCCC 240

AGTTCACTTG GGAGATTGCA GCTTTGAGGG TTGGATAGTA GGAAATCCTG CCTGTACTAG 300

CAACTTTGGA ATCAGAGAGT GGTCATATTT GATTGAAGAC CCATCTGCAC CTCATGGACT 360

GTGCTACCCA GGAGAACTGG ACAACAATGG AGAATTGAGG CATCTGTTCA GTGGAATCAA 420

ATCGTTTAGC AGAACTGAAT TGATCGCACC TACTTCTTGG GGAGAAGTGA ATGATGGAGC 480

AACATCTGCC TGCAGGGACA ATACGGGGAC AAGCAGTTTC TATCGAAATC TAGTATGGTT 540

TGTGAAGAGA GGTAACAAAT ATCCAGTAAT CAGCAGGACT TACAACAACA CAACTGGTAG 600

AGATGTTTTA GTTATATGGG GGATACACCA TCCAGTCTCA ACAGACGAGA CCAAATCATT 660

ATATGTCAAT AGTGATCCTT ATACGCTAGT GTCTACCAGC TCGTGGAGTA AAAAATACAA 720

ATTGGAAACT GGAGTCAGGC CTGGGTACAA TGGTCAAAGG AGTTGGATGA AGATTTACTG 780

GGTCCTAATG CATCCAGGAG AATCAATAAC CTTTGAAAGC AATGGAGGAT TGTTGGCCCC 840

AAGATATGGT TATATAATCG AGGAGTATGG AAAAGGAAGA ATCTTCCAAA GCCCAATCAG 900

AGTCGCCAGA TGCAACACCA AATGCCAGAC TTCTGTCGGA GGGATAAACA CCAACAAAAC 960

ATTCCAGAAC ATAGAGAGAA ATGCACTTGG AAATTGCCCC AAGTACATAA AATCAGGGCA 1020

GCTTAAGTTG GCAACTGGGC TTAGGAATGT ACCTTCCACA TCAAACAGAG GACTGTTTGG 1080

GGCGATTGCA GGGTTCATAG AAGGTGGTTG GCCAGGATTA ATAAATGGTT GGTACGGATT 1140

TCAACATCAG AATGAACAAG GGGTTGGAAT CGCTGCAGAT AAAGAATCGA CACAAAAGGC 1200

CATTGACCAG ATAACAACAA AGATAAATAA CATCATTGAC AAAATGAACG GGAATTATGA 1260

CTCTATACGA GGTGAATTCA GCCAAGTAGA ACGACGAATA AACATGCTTG CAGACAGAAT 1320

AGATGACGCT GTGACTGATG TGTGGTCATA CAATGCTAAA CTTCTTGTGC TACTGGAAAA 1380

TGACAAAACC CTAGACATGC ATGACGCCAA TGTCAGAAAT TTGCATGAGC AAGTGCGAAG 1440

AACACTAAAG GCCAATGCAA TTAATGAAGG AAATGGGTGC TTTGAGCTCC TTCATAAATG 1500

TAATGACTCT TGTATGGAGA CAATAAGAAA CGGGACTTAC AACCATGCTG AATATGCAGA 1560

AGAGTCGAAA CTGAAGAGAC AAGAAATAGA GGGTATAAAG CTGAAGTCAG AAGACAATGT 1620

TTACAGAGCA TTGTCAATAT ACAGTTGCAT TGCAAGCAGT GTTGTGCTAG TAGGGCTCAT 1680

ACTTGCATTT ATAATGTGGG CATGTAGTAG TGGCAATTGC CGGTTCAATG TTTGTATATA 1740

AATAGAAAAA AACACCCTTG TTTCTACT 1768

**A_seg4_H14**

AGCAAAAGCA GGGGAAAATG ATTGCACTCA TATTGGTTGC ACTGGCTCTG AGCCACACTG 60

CTTATTCTCA GATCACAAAT GGGACAACAG GAAACCCCAT TATATGCTTG GGGCATCATG 120

CAGTGGAAAA CGGCACATCT GTTAAAACAC TAACAGACAA TCACGTAGAA GTTGTGTCAG 180

CTAAAGAATT AGTTGAGACG AACCACACTG ATGAACTGTG CCCAAGCCCC TTGAAGCTTG 240

TCGACGGGCA AGACTGCCAC CTCATCAATG GTGCATTGGG GAGTCCAGGC TGTGACCGTT 300

TGCAGGACAC CACTTGGGAT GTCTTCATTG AAAGGCCCAC TGCAGTAGAC ACATGTTATC 360

CATTCGACGT CCCAGATTAC CAGAGTCTCA GAAGCATCCT AGCAAGCAGT GGGAGTTTGG 420

AGTTCATCGC CGAACAATTC ACCTGGAATG GTGTCAAAGT TGACGGATCA AGCAGTGCTT 480

GTTTGAGGGG CGGTCGCAAC AGCTTCTTCT CCCGACTAAA CTGGCTAACC AAAGCAACAA 540

ATGGAAACTA TGGACCTATT AACGTCACTA AAGAAAATAC GGGCTCTTAT GTCAGGCTCT 600

ATCTCTGGGG AGTGCATCAC CCATCAAGCG ATAATGAGCA AACGGATCTC TACAAGGTGG 660

CAACAGGGAG AGTAACAGTA TCTACCCGCT CGGACCAAAT CAGTATTGTT CCCAATATAG 720

GAAGTAGACC GAGGGTAAGG AATCAGAGCG GCAGGATAAG CATCTACTGG ACCCTAGTAA 780

ACCCAGGGGA CTCCATCATT TTCAACAGTA TTGGGAATTT GATTGCACCA AGAGGCCACT 840

ACAAAATAAG CAAATCTACT AAGAGCACAG TGCTTAAAAG TGACAAAAGG ATTGGGTCAT 900

GCACAAGCCC TTGCTTAACT GATAAAGGTT CGATCCAAAG TGACAAACCT TTTCAGAATG 960

TATCAAGGAT TGCTATAGGA AACTGCCCGA AATATGTAAA GCAAGGGTCC CTGATGTTAG 1020

CAACTGGAAT GCGCAACATC CCTGGCAAAC AGGCAAAGGG CTTATTTGGG GCAATTGCTG 1080

GATTCATTGA AAATGGTTGG CAAGGCCTGA TTGATGGGTG GTATGGATTC AGGCACCAAA 1140

ATGCTGAAGG AACAGGAACT GCTGCAGACC TGAAGTCAAC TCAGGCAGCC ATTGATCAGA 1200

TAAATGGCAA GCTGAACAGA TTGATAGAGA AGACAAATGA AAAATATCAC CAAATAGAAA 1260

AGGAATTCGA ACAGGTGGAA GGAAGAATAC AAGACCTTGA GAAGTACGTT GAGGACACTA 1320

AGATTGATTT GTGGTCATAC AATGCTGAAT TGCTAGTAGC ACTAGAGAAT CAGCACACAA 1380

TAGATGTCAC AGACTCCGAA ATGAACAAGC TTTTTGAAAG AGTAAGAAGG CAATTAAGAG 1440

AGAATGCAGA AGATCAAGGC AACGGTTGTT TCGAGATATT CCATCAGTGT GACAACAATT 1500

GTATAGAAAG CATTAGAAAC GGAACTTATG ACCACAACAT CTACAGGGAT GAAGCCATCA 1560

ACAATCGAAT CAAAATAAAT CCTGTCACTT TGACGATGGG GTACAAGGAC ATAATCCTGT 1620

GGATTTCTTT CTCCATGTCA TGCTTTGTCT TCGTGGCACT GATTCTGGGA TTTGTTCTAT 1680

GGGCTTGTCA AAACGGGAAT ATCCGATGCC AAATCTGTAT ATAAAGAAAA AACACCCTTG 1740

TTTCTACT 1748

**A_seg4_H15**

AGCAAAAGCA GGGGAAACAA AATGAACACT CAAATCATTG TCATTCTAGT CCTCGGACTG 60

TCAATGGTGA AATCTGACAA GATTTGTCTC GGGCACCATG CCGTAGCAAA TGGGACAAAA 120

GTCAACACAC TAACTGAGAG AGGAGTGGAA GTGGTCAATG CCACGGAGAC AGTGGAGATT 180

ACCGGAATAG ATAAAGTGTG CACAAAAGGG AAGAAAGCAG TGGACCTGGG GTCTTGTGGA 240

ATACTGGGAA CTATCATTGG GCCTCCACAA TGTGATCTTC ATCTTGAATT CAAAGCTGAT 300

CTGATAATAG AAAGAAGAAA TTCAAGTGAC ATCTGTTACC CAGGAAGATT CACTAATGAG 360

GAAGCACTGA GACAAATAAT CAGAGAATCT GGAGGAATTG ACAAAGAGTC AATGGGCTTT 420

AGATATTCAG GAATAAGAAC AGACGGGGCA ACCAGTGCGT GTAAGAGAAC AGTGTCCTCT 480

TTCTACTCAG AAATGAAATG GCTTTCATCC AGCATGAATA ACCAGGTGTT CCCACAACTG 540

AATCAGACAT ACAGGAACAC CAGAAAAGAA CCAGCCCTAA TTGTCTGGGG AGTACATCAT 600

TCAAGTTCCT TGGATGAGCA AAATAAGCTA TATGGAACTG GGAACAAGCT GATAACAGTA 660

GGAAGCTCAA AGTACCAACA ATCGTTTTCA CCAAGTCCAG GGGCCAGGCC CAAAGTGAAT 720

GGTCAGGCCG GGAGGATCGA CTTTCATTGG ATGCTATTGG ACCCAGGGGA TACAGTCACT 780

TTTACCTTCA ATGGTGCATT CATAGCCCCA GATAGAGCCA CCTTTCTCCG CTCTAATGCC 840

CCTTCAGGAA TTGAGTACAA TGGGAAGTCA CTGGGAATAC AGAGTGATGC ACAAATCGAT 900

GAATCATGTG AAGGGGAATG CTTCTACAGT GGAGGGACAA TAAACAGCCC TTTACCATTT 960

CAAAACATCG ATAGTAGGGC TGTCGGAAAG TGCCCCAGAT ATGTGAAGCA ATCAAGCTTG 1020

CCGCTGGCCT TAGGAATGAA AAATGTACCA GAGAAAATAC GTACTAGGGG ACTGTTCGGT 1080

GCAATTGCAG GATTCATCGA AAATGGATGG GAAGGGCTCA TTGATGGATG GTATGGATTT 1140

AGGCATCAGA ATGCACAAGG GCAGGGAACA GCTGCTGACT ACAAGAGTAC TCAGGCTGCA 1200

ATTGACCAGA TAACAGGGAA ACTTAATAGG TTAATTGAAA AAACCAACAA ACAGTTTGAA 1260

CTCATAGACA ATGAGTTCAC TGAAGTGGAG CAGCAGATAG GCAATGTAAT AAACTGGACA 1320

AGGGACTCCT TGACTGAGAT CTGGTCATAC AATGCCGAAC TGCTAGTAGC AATGGAGAAT 1380

CAGCATACAA TTGACCTTGC AGATTCTGAA ATGAACAAAC TCTATGAGAG AGTGAGAAGA 1440

CAGCTAAGGG AGAATGCCGA GGAGGATGGA ACTGGATGTT TTGAGATTTT CCACCGATGT 1500

GACGATCAAT GTATGGAGAG CATACGGAAT AATACTTACA ATCACACTGA ATATCGACAG 1560

GAAGCCTTAC AAAATAGGAT AATGATCAAT CCGGTAAAGC TTAGTAGTGG GTACAAAGAT 1620

GTGATACTAT GGTTTAGCTT CGGGGCATCA TGTGTAATGC TTCTAGCCAT TGCTNTGGGT 1680

CTTATTTTCA TGTGTGTGAA AAACGGGAAT CTGCGGTGCA CTATCTGTAT ATAATTATTT 1740

GAAAAAACAC CCTTGTTTCT ACT 1763

**A_seg4_H16**

AGCAAAAGCA GGGGATATTG TCAAAACAAC AGAATGGTGA TCAAAGTGCT CTACTTTCTC 60

ATCGTATTGT TAAGTAGGTA TTCGAAAGCA GACAAAATAT GCATAGGATA TCTAAGCAAC 120

AACGCCACAG ACACAGTAGA CACACTGACA GAGAACGGAG TTCCAGTGAC CAGCTCAGTT 180

GATCTCGTTG AAACAAACCA CACAGGAACA TACTGCTCAC TGAATGGAAT CAGCCCAATT 240

CATCTTGGTG ACTGCAGCTT TGAGGGATGG ATCGTAGGAA ACCCTTCCTG TGCCACCAAC 300

ATCAACATCA GAGAGTGGTC GTATCTAATT GAGGACCCCA ATGCCCCCAA CAAACTCTGC 360

TTCCCAGGAG AGTTAGATAA TAATGGAGAA TTACGACATC TCTTCAGCGG AGTGAACTCT 420

TTTAGCAGAA CAGAATTAAT AAGTCCCAAC AAATGGGGAG ACATTCTGGA TGGAGTCACC 480

GCTTCTTGCC GCGATAATGG GGCAAGCAGT TTTTACAGAA ATTTGGTCTG GATAGTGAAG 540

AATAAAAATG GAAAATACCC TGTCATAAAG GGGGATTACA ATAACACAAC AGGCAGAGAT 600

GTTCTAGTAC TCTGGGGCAT TCACCATCCG GATACAGAAA CAACAGCCAT AAACTTGTAC 660

GCAAGCAAAA ACCCCTACAC ATTAGTATCA ACAAAGGAAT GGAGCAAAAG ATATGAACTA 720

GAAATTGGCA CCAGAATAGG TGATGGACAG AGAAGTTGGA TGAAACTATA TTGGCACCTC 780

ATGCGCCCTG GAGAGAGGAT AATGTTTGAA AGCAACGGGG GCCTTATAGC GCCCAGATAC 840

GGATACATCA TTGAGAAGTA CGGTACAGGA CGAATTTTCC AAAGTGGAGT GAGAATGGCC 900

AAATGCAACA CAAAGTGTCA AACATCATTA GGTGGGATAA ACACCAACAA AACTTTCCAA 960

AACATAGAGA GAAATGCTCT TGGAGATTGC CCAAAGTACA TAAAGTCTGG ACAGCTGAAG 1020

CTTGCAACTG GGCTGAGAAA TGTCCCATCC GTTGGTGAAA GAGGTTTGTT TGGTGCAATT 1080

GCAGGCTTCA TAGAAGGAGG GTGGCCTGGG CTAATTAATG GATGGTATGG TTTCCAGCAT 1140

CAGAATGAAC AGGGGACTGG CATTGCTGCA GACAAAGCCT CCACTCAGAA AGCGATAGAT 1200

GAAATAACAA CAAAAATTAA CAATATAATA GAGAAGATGA ACGGAAACTA TGATTCAATA 1260

AGAGGGGAAT TCAATCAAGT AGAAAAGAGG ATCAACATGC TCGCTGATCG AGTTGATGAT 1320

GCAGTAACTG ACATATGGTC GTACAATGCT AAACTTCTTG TACTGCTTGA AAATGGGAGA 1380

ACATTGGACT TACACGACGC AAATGTCAGG AACTTACACG ATCAGGTCAA GAGAATATTG 1440

AAAAGTAATG CTATTGATGA AGGAGATGGT TGCTTCAATC TTCTTCACAA ATGTAATGAC 1500

TCATGCATGG AAACTATTAG AAATGGGACC TACAATCATG AAGATTACAG GGAAGAATCA 1560

CAACTGAAAA GGCAGGAAAT TGAGGGAATA AAATTGAAGT CTGAAGACAA TGTGTATAAA 1620

GTACTGTCGA TTTATAGCTG CATTGCAAGC AGTATTGTGC TGGTAGGTCT CATACTTGCG 1680

TTCATAATGT GGGCATGCAG CAATGGAAAT TGCCGGTTTA ATGTTTGTAT ATAGTCGGAA 1740

AAAATACCCT TGTTTCTACT 1760

**A_seg5_NP**

AGCAAAAGCA GGGTTAATAA TCACTCACTG AGTGACATCA AAATCATGGC GTCCCAAGGC 60

ACCAAACGGT CTTATGAACA GATGGAAACT GATGGGGATC GCCAGAATGC AACTGAGATT 120

AGGGCATCCG TCGGGAAGAT GATTGATGGA ATTGGGAGAT TCTACATCCA AATGTGCACT 180

GAACTTAAAC TCAGTGATCA TGAAGGGCGG TTGATCCAGA ACAGCTTGAC AATAGAGAAA 240

ATGGTGCTCT CTGCTTTTGA TGAAAGAAGG AATAAATACC TGGAAGAACA CCCCAGCGCG 300

GGGAAAGATC CCAAGAAAAC TGGGGGGCCC ATATACAGGA GAGTAGATGG AAAATGGATG 360

AGGGAACTCG TCCTTTATGA CAAAGAAGAA ATAAGGCGAA TCTGGCGCCA GGCCAACAAT 420

GGTGAGGATG CGACAGCTGG TCTAACTCAC ATAATGATCT GGCATTCCAA TTTGAATGAT 480

GCAACATACC AGAGGACAAG AGCTCTTGTT CGAACTGGAA TGGATCCCAG AATGTGCTCT 540

CTGATGCAGG GCTCGACTCT CCCTAGAAGG TCCGGAGCTG CAGGTGCTGC AGTCAAAGGA 600

ATCGGGACAA TGGTGATGGA ACTGATCAGA ATGGTCAAAC GGGGGATCAA CGATCGAAAT 660

TTCTGGAGAG GTGAGAATGG GCGGAAAACA AGAAGTGCTT ATGAGAGAAT GTGCAACATT 720

CTTAAAGGAA AATTTCAAAC AGCTGCACAA AGAGCTATGG TGGATCAAGT GAGAGAAAGT 780

CGGAACCCAG GAAATGCTGA GATCGAAGAT CTCATATTTT TGGCAAGATC TGCATTGATA 840

TTGAGAGGGT CAGTTGCTCA CAAATCTTGC CTACCTGCCT GTGCGTATGG ACCTGCAGTA 900

TCCAGTGGGT ACGACTTCGA AAAAGAGGGA TATTCCTTGG TGGGAATAGA CCCTTTCAAA 960

CTACTTCAAA ATAGCCAAAT ATACAGCCTA ATCAGACCTA ACGAGAATCC AGCACACAAG 1020

AGTCAGCTGG TGTGGATGGC ATGCCATTCT GCTGCATTTG AAGATTTAAG ATTGTTAAGC 1080

TTCATCAGAG GGACAAAAGT ATCTCCTCGG GGGAAACTGT CAACTAGAGG AGTACAAATT 1140

GCTTCAAATG AGAACATGGA TAATATGGGA TCGAGCACTC TTGAACTGAG AAGCGGGTAC 1200

TGGGCCATAA GGACCAGGAG TGGAGGAAAC ACTAATCAAC AGAGGGCCTC CGCAGGCCAA 1260

ACCAGTGTGC AACCTACGTT TTCTGTACAA AGAAACCTCC CATTTGAAAA GTCAACCATC 1320

ATGGCAGCAT TCACTGGAAA TACGGAGGGA AGAACTTCAG ACATGAGGGC AGAAATCATA 1380

AGAATGATGG AAGGTGCAAA ACCAGAAGAA GTGTCATTCC GGGGGAGGGG AGTTTTCGAG 1440

CTCTCAGACG AGAAGGCAAC GAACCCGATC GTGCCCTCTT TTGATATGAG TAATGAAGGG 1500

TCTTATTTCT TCGGAGACAA TGCAGAAGAG TACGACAATT AAGGAAAAAT ACCCTTGTTT 1560

CTACT 1565

**A_seg6_NA1**

AGCAAAAGCA GGAGTTTAAA ATGAATCCAA ATCAAAAAAT AATAACCATT GGATCAATCA 60

GTATAGCAAT CGGAATAATT AGTCTAATGT TGCAAATAGG AAATATTATT TCAATATGGG 120

CTAGTCACTC AATCCAAACT GGAAGTCAAA ACCACACTGG AATATGCAAC CAAAGAATCA 180

TCACATATGA AAACAGCACC TGGGTGAATC ACACATATGT TAATATTAAC AACACTAATG 240

TTGTTGCTGG AAAGGACAAA ACTTCAGTGA CATTGGCCGG CAATTCATCT CTTTGTTCTA 300

TCAGTGGATG GGCTATATAC ACAAAAGACA ACAGCATAAG AATTGGCTCC AAAGGAGATG 360

TTTTTGTCAT AAGAGAACCT TTCATATCAT GTTCTCACTT GGAATGCAGA ACCTTTTTTC 420

TGACCCAAGG TGCTCTATTA AATGACAAAC ATTCAAATGG AACCGTTAAG GACAGAAGTC 480

CTTATAGGGC CTTAATGAGC TGTCCTCTAG GTGAAGCTCC GTCCCCATAC AATTCAAAGT 540

TTGAATCAGT TGCATGGTCA GCAAGCGCAT GCCATGATGG CATGGGCTGG TTAACAATCG 600

GAATTTCTGG TCCAGACAAT GGAGCTGTGG CTGTACTAAA ATACAACGGA ATAATAACTG 660

AAACCATAAA AAGTTGGAAA AAGCGAATAT TGAGAACACA AGAGTCTGAA TGTGTCTGTG 720

TGAACGGGTC ATGTTTCACC ATAATGACCG ATGGCCCGAG TAATGGGGCC GCCTCGTACA 780

AAATCTTCAA GATCGAAAAG GGGAAGGTTA CTAAATCAAT AGAGTTGAAT GCACCCAATT 840

TTCATTATGA GGAATGTTCC TGTTACCCAG ACACTGGCAC AGTGATGTGT GTATGCAGGG 900

ACAACTGGCA TGGTTCAAAT CGACCTTGGG TGTCTTTTAA TCAAAACTTG GATTATCAAA 960

TAGGATACAT CTGCAGTGGA GTGTTCGGTG ACAATCCGCG TCCCAAAGAT GGAAAGGGCA 1020

GCTGTAATCC AGTGACTGTT GATGGAGCAG ACGGAGTTAA GGGGTTTTCA TACAAATATG 1080

GTAATGGTGT TTGGATAGGA AGGACTAAAA GTAACAGACT TAGAAAGGGG TTTGAGATGA 1140

TTTGGGATCC TAATGGATGG ACAGATACCG ACAGTGATTT CTCAGTGAAA CAGGATGTTG 1200

TGGCAATAAC TGATTGGTCA GGGTACAGCG GAAGTTTCGT TCAACATCCT GAGTTAACAG 1260

GATTGGACTG TATAAGACCT TGCTTCTGGG TTGAGTTAGT CAGAGGACTG CCTAGAGAAA 1320

ATACAACAAT CTGGACTAGT GGGAGCAGCA TTTCTTTTTG TGGCGTTGAT AGTGATACTG 1380

CAAACTGGTC TTGGCCAGAC GGTGCTGAGT TGCCGTTCAC CATTGACAAG TAGTTCGTTG 1440

AAAAAAAACT CCTTGTTTCT ACT 1463

**A_seg6_NA2**

AGCAAAAGCA GGAGTAAAGA TGAATCCAAA TCAAAAGATA ATAACGATTG GCTCTGTTTC 60

TCTCACCATT TCCACAATAT GCTTCTTCAT GCAAATTGCC ATCTTGATAA CCACTGTAAC 120

ATTGCATTTC AAGCAATATG AATTCAACTC CCCCCCAAAC AACCAAGTGA TGCTGTGTGA 180

ACCAACAATA ATAGAAAGAA ACATAACAGA GATAGTGTAC CTGACCAACA CCACCATAGA 240

GAAGGAAATA TGCCCCAAAC TAGCAGAATA CAGAAATTGG TCAAAGCCGC AATGTGACAT 300

TACAGGATTT GCACCTTTTT CTAAGGACAA TTCGATTAGG CTTTCCGCTG GTGGGGACAT 360

CTGGGTGACA AGAGAACCTT ATGTGTCATG CGATCCTGAC AAGTGTTATC AATTTGCCCT 420

TGGACAGGGA ACAACACTAA ACAACGTGCA TTCAAATGAC ACAGTACATG ATAGGACCCC 480

TTATCGGACC CTATTGATGA ATGAGTTAGG TGTTCCATTT CATCTGGGGA CCAAGCAAGT 540

GTGCATAGCA TGGTCCAGCT CAAGTTGTCA CGATGGAAAA GCATGGCTGC ATGTTTGTGT 600

AACGGGGGAT GATAAAAATG CAACTGCTAG CTTCATTTAC AATGGGAGGC TTGTAGATAG 660

TATTGTTTCA TGGTCCAAAG AAATCCTCAG GACCCAGGAG TCAGAATGCG TTTGTATCAA 720

TGGAACTTGT GCAGTAGTAA TGACTGATGG GAGCGCTTCA GGAAAAGCTG ATACTAAAAT 780

ACTATTCATT GAGGAGGGGA AAATCGTTCA TACTAGCAAA TTGTCAGGAA GTGCTCAGCA 840

TGTCGAGGAG TGCTCCTGCT ATCCTCGATA TCCTGGTATC AGATGTGTCT GCAGAGACAA 900

CTGGAAAGGC TCCAATAGGC CCATCGTAGA TATAAACATA AAGGATTATA GCATTGTTTC 960

CAGTTATGTG TGCTCAGGAC TTGTTGGAGA CACACCCAGA AAAAACGACA GCTCCAGCAG 1020

TAGCCATTGC TTGGATCCTA ACAATGAAGA AGGTGGTCAT GGAGTGAAAG GCTGGGCCTT 1080

TGATGATGGA AATGACGTGT GGATGGGAAG AACGATCAGC GAGAAGTTAC GCTCAGGATA 1140

TGAAACCTTC AAAGTCATTG AAGGCTGGTC CAACCCTAAT TCCAAATTGC AGATAAATAG 1200

GCAAGTCATA GTTGATAGAG GTAATAGGTC CGGTTATTCT GGTATTTTCT CTGTTGAAGG 1260

CAAAAGCTGC ATCAATCGGT GCTTTTATGT GGAGTTGATA AGGGGAAGAA AAGAGGAAAC 1320

TGAAGTCTTG TGGACCTCAA ACAGTATTGT TGTGTTTTGT GGCACCTCAG GTACATATGG 1380

AACAGGCTCA TGGCCTGATG GGGCGGACAT CAATCTCATG CCTATATAAG CTTTCGCAAT 1440

TTTAGAAAAA AACTCCTTGT TTCTACT 1467

**A_seg6_NA3**

AGCAAAAGCA GGTGCGAGAT GAATCCAAAT CAGAAGATAA TAACAATTGG GGTAGTGAAT 60

ACTACTCTAT CAACAATAGC CCTTCTCATC GGAGTGGGAA ATTTGGTTTT CAACACAGTC 120

ATACATGAGA AAATAGGGGA CCACCAAACA GTGATTCACC CAACAATAAT GACCCCTGCA 180

GTACCAAACT GCAGTGACAC TATAATAACA TATAATAACA CTGTGATAAA CAACATAACA 240

ACAACAATAA TAACTGAAGC GGAAAGGCTT TTCAAGCCTC CACTGCCACT GTGCCCCTTC 300

CGAGGATTCT TCCCTTTTCA CAAGGACAAT GCAATACGAT TGGGTGAGAA CAAGGACGTC 360

ATAGTCACAA GAGAGCCTTA TGTTAGCTGC GATAATAACA ATTGCTGGTC CTTTGCTCTC 420

GCGCAAGGAG CACTGTTAGG GACTAAACAT AGCAATGGAA CCATTAAAGA CAGGACACCA 480

TATAGGTCTC TAATCCAATT CCCAATGGGA ACAGCCCCAG TATTGGGAAA TTACAAGGAG 540

ATATGCATTG CTTGGTCGAG TAGCAGTTGC TTTGATGGGA AAGAGTGGAT GCATGTATGC 600

ATGACAGGGA ATGATAATGA TGCAAGTGCT CAAATAATAT ACGCAGGGAG AATGACAGAC 660

TCCATCAAAT CGTGGAGAAG GGACATATTA AGAACCCAAG AGTCCGAATG TCAATGCATT 720

AGCGGAACTT GTGTTGTTGC TGTTACAGAT GGCCCTGCTG CTAATAGCGC AGATCACAGG 780

GTTTATTGGA TACGAGAGGG AAGAATAGTG AAGTATGAAA ATGTCCCTAA AACAAAGATA 840

CAACACTTAG AAGAGTGTTC CTGCTATGTG GACATCGATG TGTACTGTAT ATGCAGGGAC 900

AATTGGAAGG GTTCCAACAG GCCTTGGATG AGAATCAACA ACGAGACTAT ACTGGAGACA 960

GGATATGTAT GCAGTAAATT TCATTCGGAC ACTCCCAGGC CAGCTGACCC CTCAACAGTA 1020

TCATGTGACT CCCCAAGCAA CATTAATGGA GGACCCGGAG TCAAGGGATT TGGTTTCAAA 1080

GTCGGCAATG ATGTATGGTT GGGCAGAACA GTGTCAACTA GTGGTAGGTC GGGCTTTGAA 1140

ATCATCAAAG TCACAGAGGG GTGGATCAAC TCTCCCAATC ATGCCAAGTC AATTACACAA 1200

ACACTGGTGT CCAACAATGA TTGGTCAGGC TATTCAGGTA GTTTCATTGT CAAAACCAAG 1260

GACTGTTTTC AGCCCTGTTT TTATGTCGAG CTTATACGAG GGAGACCCAA CAAGAATGAT 1320

GATGTCTCTT GGACAAGCAA TAGTATAGTT ACTTTCTGTG GATTAGACAA TGAACCTGGA 1380

TCGGGAAATT GGCCAGATGG TTCCAACATT GGGTTTATGC CCAAGTAACA GAAAAAAGCA 1440

CCTTGTTTCT ACT 1453

**A_seg6_NA4**

AGCAAAAGCA GGAGTTTCAT AATGAATCCA AATCAGAAAA TCATAACCAT CGGCAGTGTT 60

AGCATTATAT TAACGACGAT AGGCCTTCTC CTCCAAATAA CAAGTTTATG CTCAATATGG 120

TTTAGCCATT ATAACCAGGT GACACAGACA CATGAACAAC CTTGTTCCAA CAACACAACA 180

AATTATTACA ATGAGACTTT TGTTAATGTC ACCAATGTGC AGAACAATTA TACCACAGTA 240

ATTGAGTCCT CAGCCCCTGA TGTTGTTCAC TACTCTAGTG GAAGAGACTT GTGCCCAATA 300

AGGGGATGGG CACCTCTGAG TAAGGACAAT GGGATTAGAA TTGGATCCCG AGGCGAAGTA 360

TTTGTCATAC GGGAGCCTTT CATATCATGC TCCATTAGTG AATGCAGGAC CTTTTTCTTA 420

ACTCAGGGAG CCCTTCTCAA TGACAAACAC TCAAATGGGA CAGTAAAAGA CCGGAGTCCC 480

TTCCGCACAT TAATGAGTTG TCCCATAGGA GTTGCCCCCT CTCCTAGCAA TAGCCGCTTT 540

GAATCTGTGG CATGGTCTGC TACTGCATGT AGTGACGGAC CCGGTTGGCT AACACTAGGG 600

ATCACTGGCC CAGATGCTAC TGCTGTAGCA GTGCTGAAAT ACAATGGTAT AATAACAGAC 660

ACATTAAAAA GCTGGAAGGG AAATATTATG CGAACACAAG AGTCCGAATG CGTGTGTCAG 720

GATGAATTTT GTTATACTCT GATAACAGAC GGACCATCTG ACGCACAAGC TTTCTATAAG 780

ATACTAAAAA TCAGGAAAGG GAAAATAGTA AGTATGGAAG ATGTGGATGC AACAGGGTTC 840

CATTTCGAAG AATGTTCCTG TTATCCGAGC GGGACAGATA TTGAGTGTGT ATGTCGGGAC 900

AATTGGCGGG GAAGCAATCG ACCATGGATA AGATTCAACA GTAATCTTGA TTACCAAATC 960

GGCTATGTAT GTAGTGGGAT ATTTGGGGAC AATCCCAGGC CCATGGATGG CACAGGCTCA 1020

TGTAATAGCC CAGTCAATAA TGGGAAGGGA AGATATGGAG TGAAGGGGTT CAGCTTTAGG 1080

TATGGGGATG GTGTCTGGAT AGGAAGGACA AAGAGCTTGG AATCCAGAAG CGGCTTTGAA 1140

ATGGTGTGGG ATGCTAATGG ATGGGTGTCG ACAGACAAGG ATTCAAATGG TGTACAGGAC 1200

ATTATAGACA ACGACAATTG GTCTGGTTAC AGCGGGAGTT TCAGCATTAG AGGTGAGACA 1260

ACAGGCAGGA ATTGCACTGT CCCATGTTTC TGGGTTGAAA TGATAAGAGG GCAGCCTAAA 1320

GAAAAGACTA TATGGACCAG TGGTAGTAGT ATTGCGTTCT GTGGTGTTAA TTCTGATACC 1380

ACAGGTTGGT CATGGCCCGA TGGCGCTCTG CTGCCCTTTG ACATAGACAA GTGATTTTTC 1440

GAAAAAAACT CCTTGTTTCT ACT 1463

**A_seg6_NA5**

AGCAAAAGCA GGAGTTTAAA ATGAATCCAA ATCAGAAAAT AATAACAATT GGCTCTGTGT 60

CGTTAGCACT AGTTATATTC AACATACTGC TTCATATTGC ATCAATAGTC ATAGGAATAA 120

TATCAGTGAC AAAAGAAAGC AGTGTGTCAT CATCCTGCAA CACCACTGAG ATTTACAATG 180

AAACTGTAAG GCTGGAAACT ATAACAATTC CTATCAATAA CACTGTGTAT ATAGAAAGGG 240

AGTTACGTCA AGAACCCGAT TTCTTAAACA ACACAGAACC TCTCTGCAAT GTATCCGGGT 300

TTGCAATAGT TTCCAAGGAC AATGGAATCA GAATAGGGTC AAGGGGACAC GTGTTTGTCA 360

TAAGAGAACC ATTTGTGGCA TGTGGCCCCA CAGAATGTAG AACATTTTTC CTAACGCAAG 420

GTGCCTTACT GAATGATAAA CATTCCAACA ATACAGTGAA AGACAGAAGT CCTTATCGTG 480

CATTGATGAG TGTGCCATTA GGATCTTCAC CCAATGCCTA CCAGGCCAAG TTTGAGTCTG 540

TTGCATGGTC GGCCACAGCA TGCCATGATG GTAAAAAATG GCTGGCAGTA GGGATAAGTG 600

GTGCGGATGA CGATGCTTAT GCTGTAATTC ATTATGGGGG AATGCCAACA GATGTGGTGA 660

GATCATGGAG AAAGCAAATT CTAAGAACAC AAGAATCGTC ATGTGTATGT ATGAATGGGA 720

ACTGTTATTG GGTAATGACA GATGGTCCTG CGAACAAACA GGCTAGTTAC AAGATTTTCA 780

AGTCTCGTGA GGGAATGGTG ACAAATGAAA GAGAAGTGTC GTTTCAGGGA GGTCACATTG 840

AAGAGTGTTC TTGCTATCCC AACTTGGGCA AAGTGGAATG TGTTTGCCGG GATAATTGGA 900

ATGGAATGAA TAGACCAATT TTGGTTTTTG ATGAGGACCT GGACTATGAG GTGGGTTATT 960

TATGTGCTGG AATACCGACA GACACTCCAC GGGTTCAGGA CAGTAGTTTC ACTGGTTCCT 1020

GCACAAATGC TGTTGGAGGG AGTGGGACGA ATAACTATGG AGTGAAGGGA TTTGGCTTCA 1080

GACAAGGTAA TAGTGTGTGG GCAGGAAGAA CAGTCAGCAT TTCGTCCCGA AGTGGTTTTG 1140

AAATCCTATT AATAGAGGAC GGTTGGATTA AAACAAGCAA AACAATCGCC AAAAAGGTGG 1200

AGGTCCTCAA CAACAAGAAT TGGTCAGGAT ACAGCGGAGC TTTCACCATC CCAACCACAA 1260

TGACTGGTAA ACAATGCTTA GTTCCATGTT TCTGGCTGGA AATGATAAGA GGAAAACCAG 1320

AAGAGAGGAC AAGCATTTGG ACCTCTAGTA GCTCCACAGT ATTTTGTGGT GTTTCAAGTG 1380

AGGTCCCAGG ATGGTCCTGG GATGATGGAG CAATTCTTCC CTTTGACATC GATAAGATGT 1440

AATTTGTGAA AAAAACTCCT TGTTTCTACT 1470

**A_seg6_NA6**

AGCAAAAGCA GGGTGAAAAT GAATCCAAAT CAGAAGATAA TATGCATTTC TGCCACAGGA 60

ATGACACTAT CGGTAGTAAG CCTGCTGATA GGAATTGCCA ATTTAGGCCT AAACATCGGA 120

CTCCATTATA AGGTGGGCAA TACACCAGAT GCGAACATTC CAAACATGAA CGAGACCAAT 180

TCAACAACAA CAGTAATTAA CAATAATACT CAGAATAATT TCACAAATAT CACTAACATT 240

ATATTGAACA AAAACGAGGA GAGGACATTT CTAAACCTAA CTAAGCCTCT ATGCGAAGTA 300

AACTCATGGC ACATCCTGTC AAAAGACAAT GCAATAAGAA TAGGAGAGGA CGCTCATGTG 360

CTAGTCACAA GGGAGCCCTA CTTGTCCTGC GATCCCCAAG GCTGCAGAAT GTTTGCTTTG 420

AGCCAAGGCA CAACACTCAG AGGGCGGCAT GCGAATGGGA CTATACATGA TAGGAGCCCG 480

TTCAGAGCCC TCATAAGCTG GGAAATGGGT CAAGCACCCA GCCCATATAA TGCTAGGGTC 540

GAATGCATAG GGTGGTCAAG CACGTCATGC CACGACGGCA TATCAAGAAT GTCAATATGC 600

ATGTCAGGAC CGAACAACAA TGCATCAGCA GTAGTGTGGT ACGGAGGTAG GCCAGTAACA 660

GAGATTCCAT CATGGGCAGG GAATATTCTC AGGACTCAAG AATCGGAGTG CGTATGCCAC 720

AAAGGGATCT GTCCAGTAGT CATGACAGAT GGCCCAGCAA ACAATAGGGC AGCAACTAAG 780

ATAATCTACT TCAAAGAGGG AAAGATACAA AAAATTGAAG AACTAGCAGG AAATGCCCAA 840

CATATCGAAG AATGTTCGTG CTATGGAGCA GTAAGGGTAA TCAAATGTAT ATGCAGGGAC 900

AATTGGAAGG GGGCAAATAG GCCAGTAATC ACTATAGATC CCGAAATGAT GACCCACACA 960

AGCAAGTATT TGTGCTCAAA GGTTTTAACC GATACAAGTC GCCCCAATGA TCCCACTAAT 1020

GGGAACTGTG ATGCGCCAAT AACAGGAGGG AGCCCAGATC CTGGGGTAAA AGGATTTGCA 1080

TTCCTAGATG GAGAGAACTC ATGGCTCGGA AGAACAATTA GCAAAGACTC CAGATCAGGC 1140

TATGAAATGT TAAAAGTCCC AAATGCAGAA ACTGACACCC AATCGGGGCC AATCTCACAT 1200

CAGGTGATTG TCAACAACCA AAACTGGTCG GGATACTCAG GAGCATTCAT AGACTACTGG 1260

GCAAACAAAG AGTGCTTCAA TCCTTGTTTT TATGTGGAAC TAATCAGAGG GAGGCCCAAA 1320

GAGAGTAGCG TACTGTGGAC TTCAAATAGC ATTGTAGCTC TCTGTGGATC CAAGGAGCGA 1380

TTGGGATCAT GGTCCTGGCA TGATGGTGCT GAAATCATCT ACTTTAAGTA GGAATGATTT 1440

AGGAAAAAAC ACCCTTGTTT CTACT 1465

**A_seg6_NA7**

AGCAAAAGCA GGGTGATTGA GAATGAATCC TAATCAAAAA TTATTCGCAC TCTCTGGGGT 60

GGCCATAGCA CTGAGTATCC TCAACCTACT AATAGGAATA TCCAATGTGG GACTGAATGT 120

CTCACTACAC CTAAAGGGCA GCAGTGACCA GGATAAAAAT TGGACATGCA CGAGTGTAAC 180

ACAAAACAAC ACGACTTTAA TCGAAAACAC ATATGTCAAC AATACTACTG TCATCAATAA 240

GGAAACAGGG ACTGCAAAGC AAAATTATCT AGTGCTGAAC AAAAGTTTAT GCAAAGTTGA 300

AGGATGGGTA GTGGTGGCCA AGGACAATGC CATAAGATTC GGTGAAAGTG AACAAATAAT 360

AGTGACAAGG GAGCCGTATG TGTCATGTGA TCCATTAGGA TGTAAGATGT ACGCACTGCA 420

TCAAGGGACA ACCATTAGAA ACAAGCACTC AAACGGAACA ATACACGACA GGACTGCTTT 480

CAGAGGGTTG ATATCAACTC CTTTAGGGAG CCCCCCTATA GTCAGCAATA GTGACTTTCT 540

TTGTGTAGGG TGGTCAAGCA CCAGTTGCCA TGACGGCATC GGACGGATGA CCATTTGTGT 600

GCAGGGAAAT AATGACAACG CAACAGCTAC AGTGTACTAT GACCGAAGGC TCACTACCAC 660

AATAAAAACA TGGGCAGGAA ATATCCTTAG GACGCAAGAG TCTGAATGTG TATGCCACAA 720

TGGAACATGT GTAGTAATAA TGACCGATGG ATCAGCAAGC AGCCAGGCAT ACACAAAAGT 780

TCTGTATTTT CACAAAGGAC TAGTAATAAA AGAGGAAGCC CTCAAAGGAT CAGCCAGACA 840

CATAGAGGAA TGCTCATGCT ATGGGCACAA TTCAAAAGTG ACTTGTGTAT GCAGGGACAA 900

CTGGCAAGGA GCCAATAGAC CAGTGATTGA AATAGATATG AATGCCATGG AGCATACAAG 960

CCAGTATCTA TGTACAGGGG TTCTCACTGA CACGAGTAGA CCATCAGACA AATCAATAGG 1020

AGACTGTAAT AATCCGATCA CTGGGAGTCC GGGAGCCCCT GGGGTCAAAG GATTCGGCTT 1080

CCTGGATAGT GGCAATACAT GGTTGGGCCG CACAATAAGT CCTCGTTCCA GGAGTGGTTT 1140

TGAGATGTTG AAGATACCTA ATGCTGGGAC AGACCCGAAT TCTAGAATCA CTGAGAGGCA 1200

AGAAATAGTT GACAACAACA ATTGGTCAGG ATACTCAGGA AGTTTCATTG ACTACTGGGA 1260

TGAAAGCAGT GAGTGCTACA ACCCCTGTTT TTATGTTGAA TTAATAAGAG GAAGGCCTGA 1320

AGAAGTCAAG TATGTTTGGT GGACGAGCAA CAGTTTAGTT GCACTATGTG GAAGCCCAAT 1380

CTCAGTTGGG TCCGGTTCCT TCCCCGATGG GGCACAAATC CAATACTTTT CGTAAATTGC 1440

AAAAAACACC CTTGTTTCTA CT 1462

**A_seg6_NA8**

AGCAAAAGCA GGAGTTTAAA ATGAATCCAA ATCAGAAAAT AATAACCATT GGATCAGTAT 60

CCTTAGGATT GGTAGTCCTT AATATTCTCC TACATATAGT TAGCATTACA ATAACAGTGT 120

TGGTTCTCCC TGGAAATGGA AATAATGGGA GTTGCAGTGA AACAATCATT AGGGAATACA 180

ATGAAACAGT AAGGATTGAG AAGGTAACAC AATGGCAAAA TACCAATGTC ATTGAGTATA 240

TAGAGAGACC AGAGAGTGAT CATTTCATGA ACAATACAGA ACCATTGTGT GATGCTAAGG 300

GTTTTGCACC TTTTTCCAAA GACAACGGAA TAAGAATTGG GTCGAGAGGT CATGTTTTTG 360

TTATAAGGGA ACCATTTGTT TCTTGCTCGC CAACAGAGTG CAGGACGTTC TTCCTTACTC 420

AAGGTTCCCT ACTCAATGAC AAACATTCTA ATGGCACAGT GAAAGACCGG AGCCCCTATA 480

GAACTCTAAT GAGTGTAGAA ATAGGGCAAT CACCCAATGT GTACCAGGCA AGGTTTGAGG 540

CAGTAGCGTG GTCAGCTACT GCATGTCATG ATGGGAAGAA ATGGATGACA ATTGGAGTAA 600

CGGGCCCTGA TGCCAAAGCA GTAGCAGTGG TGCATTATGG GGGAATTCCC ACTGATGTAA 660

TCAATTCCTG GGCAGGAGAT ATTCTAAGAA CTCAGGAATC ATCATGCACT TGCATTCAAG 720

GTGAGTGTTA TTGGGTAATG ACGGATGGAC CAGCAAACAG ACAAGCACAA TACAGAGCAT 780

TCAAAGCCAA ACAGGGAAAA ATAATTGGGC AAAATGAAAT CAGTTTCAAT GGAGGCCATA 840

TAGAAGAATG CTCATGCTAC CCCAATGAAG GTAAAGTGGA ATGTGTTTGT AGGGACAACT 900

GGACTGGAAC CAACAGGCCA GTATTGGTGA TTTCTCCAGA TTTGTCTTAC AGAGTCGGAT 960

ACTTATGTGC AGGTCTCCCC AGTGACACTC CAAGAGGAGA AGACAGTCAG TTCACGGGGT 1020

CATGCACTAG TCCCATGGGA AACCAGGGAT ATGGAGTTAA GGGATTTGGA TTCAGGCAGG 1080

GCAATGATGT ATGGATGGGG AGGACCATTA GCAGAACATC AAGATCAGGG TTTGAAATCC 1140

TGAAAGTCAG AAATGGCTGG GTACAAAATA GTAAGGAGCA GATCAAAAGG CAAGTTGTGG 1200

TCGATAACTT AAATTGGTCA GGATACAGTG GTTCCTTCAC ACTACCAGTG GAGTTAACAA 1260

AAAGAAATTG TCTGGTTCCA TGCTTTTGGG TTGAGATGAT AAGGGGAAAG CCAGAAGAAA 1320

AGACGATATG GACCTCAAGT AGCTCCATTG TGATGTGTGG AGTAGACCAT GAGATTGCCG 1380

ACTGGTCGTG GCACGATGGA GCTATTCTTC CTTTTGACAT CGACAAGATG TAATTTACGA 1440

AAAAAACTCC TTGTTTCTAC T 1461

**A_seg6_NA9**

AGCAAAAGCA GGGTCAAGAT GAATCCAAAT CAGAAGATTC TATGCACTTC TGCCACTGCT 60

ATCGTAATAG GCACAATTGC AGTACTCATA GGAATAGCAA ACCTAGGATT GAACATAGGA 120

CTACATCTAA AACCGAGCTG CAATTGCTCA CACTCACAAC CTGAAGCAAC CAATGCAAGC 180

CAAACAATAA TAAACAACTA CTATAATGAA ACAAACATCA CCCAAATAAG TAACACCAAC 240

ATCCAAATGG AGGAGAGAGC AAGCAGGGAA TTCAATAACT TGACTAAAGG GCTCTGTACT 300

ATAAATTCAT GGCACATATA TGGGAAAGAC AATGCGGTAA GAATTGGGGA GAATTCAGAT 360

GTTTTAGTCA CAAGAGAACC CTATGTCTCA TGTGACCCAG ATGAATGCAG GTTCTATGCT 420

CTCAGCCAAG GGACAACAAT CAGAGGGAAA CATTCAAATG GAACAATACA CGATAGGTCC 480

CAGTATCGCG CCCTGATAAG CTGGCCACTA TCATCGCCGC CCACAGTATA CAACAGCAGG 540

GTGGAATGCA TTGGATGGTC AAGTACTAGT TGCCATGATG GCAAAGCCAG GATGTCAATA 600

TGTATATCAG GGCCGAACAA CAATGCATCT GCAGTGGTCT GGTACAATAG AAGGCCTGTT 660

ACAGAAATCA ATACATGGGC CCGAAACATA CTAAGGACAC AGGAATCTGA ATGCGTATGC 720

CACAACGGTG TATGCCCGGT AGTGTTCACA GATGGGTCTG CCACTGGACC TGCAGAAACA 780

AGAATATACT ATTTCAAAGA AGGAAAAATA TTAAAATGGG AATCTCTGGC TGGAACTGCT 840

AAGCATATCG AAGAATGCTC ATGTTACGGG GAGCGAACAG GGATTACTTG CACATGCAGG 900

GATAATTGGC AGGGCTCAAA TAGACCAGTA ATTCGAATAG ATCCAGTGGC GATGACACAC 960

ACTAGTCAGT ATATATGTAG CCCTGTTCTC ACAGATAATC CCCGACCGAA TGACCCAACT 1020

GTAGGTAAGT GTAACGACCC TTATCCAGGC AATAATAACA ATGGGGTCAA AGGATTCTCA 1080

TACCTGGATG GATTTAATAC CTGGCTGGGG AGGACAATAA GCACAGCTTC AAGATCCGGA 1140

TACGAGATGC TAAAGGTGCC AAATGCATTG ACAGACGATA GGTCAAAGCC CACTCAAGGT 1200

CAGACAATCG TATTAAACAC TGACTGGAGT GGTTACAGTG GGTCCTTCAT GGACTATTGG 1260

GCTGAGGGGG ACTGCTACCG AGCGTGTTTT TACGTGGAGT TAATACGTGG AAGACCCAAG 1320

GAGGATAAAG TGTGGTGGAC CAGTAATAGT ATAGTATCGA TGTGTTCCAG CACAGAATTC 1380

CTTGGACAAT GGAACTGGCC TGATGGGGCT AAAATAGAGT ACTTCCTCTA AGATACAGAA 1440

AAAAGACCCT TGTTTCTACT 1460

**A_seg7_MP**

AGCAAAAGCA GGTAGATATT GAAAGATGAG CCTTCTAACC GAGGTCGAAA CGTATGTTCT 60

CTCTATCGTT CCATCAGGCC CCCTCAAAGC CGAGATCGCG CAGAGACTTG AAGATGTCTT 120

TGCTGGGAAA AACACAGATC TTGAGGCTCT CATGGAATGG CTAAAGACAA GACCAATTCT 180

GTCACCTCTG ACTAAGGGAA TTTTGGGGTT TGTGTTCACG CTCACCGTGC CCAGTGAGCG 240

AGGACTGCAG CGTAGACGCT TTGTCCAAAA TGCCCTCAAT GGGAATGGAG ATCCAAATAA 300

CATGGACAAA GCAGTTAAAC TGTATAGGAA ACTTAAGAGG GAGATAACGT TCCATGGGGC 360

CAAAGAAATA GCTCTCAGTT ATTCTGCTGG TGCACTTGCC AGTTGCATGG GCCTCATATA 420

CAATAGAATG GGGGCTGTAA CCACTGAAGT GGCATTTGGC CTGGTATGTG CAACATGTGA 480

ACAGATTGCT GACTCCCAGC ACAGGTCTCA TAGGCAAATG GTGGCAACAA CCAATCCATT 540

AATAAAACAT GAGAACAGAA TGGTTTTGGC CAGCACTACA GCTAAGGCTA TGGAGCAAAT 600

GGCTGGGTCA AGTGAGCAGG CAGCGGAGGC CATGGAAATT GCTAGTCAGG CCAGGCGAAT 660

GGTGCAGGCA ATGAGAGCCG TTGGGACTCA TCCTAGCTCC AGTACTGGTC TAAGAGATGA 720

TCTTCTTGAA AATTTGCAGA CCTATCAGAA ACGAATGGGG GTGCAGATGC AACGATTCAA 780

GTGACCCGCT TGTTGTTGCC GCGAGTATCA TTGGGATCTT GCACTTGATA TTGTGGATTC 840

TTGATCGTCT TTTTTTCAAA TGCGTCTATC GACTCTTCAA ACACGGCCTT AAAAGAGGCC 900

CTTCTACGGA AGGAGTACCT GAGTCTATGA GGGAAGAATA TCGAAAGGAA CAGCAGAATG 960

CTGTGGATGC TGACGACAGT CATTTTGTCA GCATAGAGTT GGAGTAAAAA ACTACCTTGT 1020

TTCTACT 1027

**A_seg8_NS**

AGCAAAAGCA GGGTGACAAA GACATAATGG ATTCCAACAC TGTGTCAAGT TTCCAGGTAG 60

ATTGCTTTCT TTGGCATATC CGGAAACAAG TTGTAGACCA AGAACTGAGT GATGCCCCAT 120

TCCTTGATCG GCTTCGCCGA GATCAGAGGT CCCTAAGAGG AAGAGGCAAT ACTCTCGGTC 180

TAGACATCAA AGCAGCCACC CATGTTGGAA AGCAAATTGT AGAAAAGATT CTGAAAGAAG 240

AATCTGATGA GGCACTTAAA ATGACCATGG TCTCCACACC TGCTTCGCGA TACATAACTG 300

ACATGACTAT TGAGGAATTG TCAAGAAACT GGTTCATGCT AATGCCCAAG CAGAAAGTGG 360

AAGGACCTCT TTGCATCAGA ATGGACCAGG CAATCATGGA GAAAAACATC ATGTTGAAAG 420

CGAATTTCAG TGTGATTTTT GACCGACTAG AGACCATAGT ATTACTAAGG GCTTTCACCG 480

AAGAGGGAGC AATTGTTGGC GAAATCTCAC CATTGCCTTC TTTTCCAGGA CATACTATTG 540

AGGATGTCAA AAATGCAATT GGGGTCCTCA TCGGAGGACT TGAATGGAAT GATAACACAG 600

TTCGAGTCTC TAAAAATCTA CAGAGATTCG CTTGGAGAAG CAGTAATGAG AATGGGGGAC 660

CTCCACTTAC TCCAAAACAG AAACGGAAAA TGGCGAGAAC AGCTAGGTCA AAAGTTTGAA 720

GAGATAAGAT GGCTGATTGA AGAAGTGAGA CACAGACTAA AAACAACTGA AAATAGCTTT 780

GAACAAATAA CATTCATGCA AGCATTACAA CTGCTGTTTG AAGTGGAACA GGAGATAAGA 840

ACTTTCTCAT TTCAGCTTAT TTAATGATAA AAAACACCCT TGTTTCTACT 890
