# Supplementary figures and images for: Concurrent circulation of avian influenza viruses H5N1 and H9N2 enhances the genetic evolution of reassortant viruses in Egyptian poultry populations
Source: PLoS One. 2026 May 8;21(5):e0348609. doi: 10.1371/journal.pone.0348609 (PMC13155612; doi:10.1371/journal.pone.0348609)

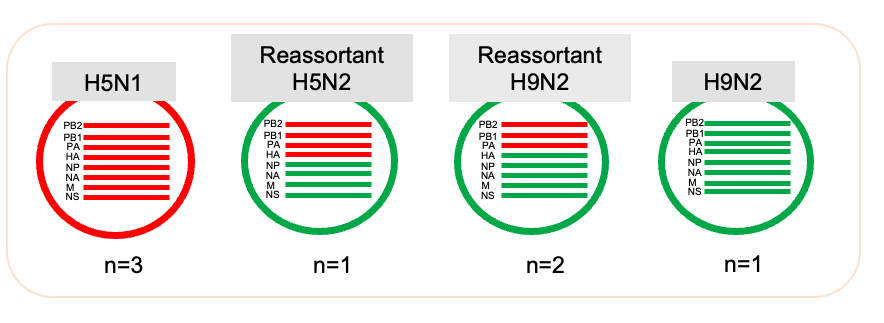

Supplement: S2 File — (PNG) [file pone.0348609.s007.png]
